# Health Equity in the Effectiveness of Web-Based Health Interventions for the Self-Care of People With Chronic Health Conditions: Systematic Review

**DOI:** 10.2196/17849

**Published:** 2020-06-05

**Authors:** Sophie Turnbull, Christie Cabral, Alastair Hay, Patricia J Lucas

**Affiliations:** 1 Centre for Academic Primary Care Population Health Sciences University of Bristol Bristol United Kingdom; 2 School for Policy Studies University of Bristol Bristol United Kingdom

**Keywords:** health equity, self-care, eHealth, intervention, diabetes, asthma, pulmonary disease, chronic obstructive, osteoarthritis

## Abstract

**Background:**

Web-based self-care interventions have the potential to reduce health inequalities by removing barriers to access to health care. However, there is a lack of evidence about the equalizing effects of these interventions on chronic conditions.

**Objective:**

This study investigated the differences in the effectiveness of web-based behavioral change interventions for the self-care of high burden chronic health conditions (eg, asthma, chronic obstructive pulmonary disease [COPD], diabetes, and osteoarthritis) across socioeconomic and cultural groups.

**Methods:**

A systematic review was conducted, following Cochrane review guidelines. We conducted searches in Ovid Medical Literature Analysis and Retrieval System Online and Cumulative Index to Nursing and Allied Health Literature databases. Studies with any quantitative design were included (published between January 1, 2006, and February 20, 2019) if they investigated web-based self-care interventions targeting asthma, COPD, diabetes, and osteoarthritis; were conducted in any high-income country; and reported variations in health, behavior, or psychosocial outcomes across social groups. Study outcomes were investigated for heterogeneity, and the possibility of a meta-analysis was explored. A narrative synthesis was provided together with a novel figure that was developed for this review, displaying heterogeneous outcomes.

**Results:**

Overall, 7346 records were screened and 18 studies were included, most of which had a high or critical risk of bias. Important study features and essential data were often not reported. The meta-analysis was not possible due to the heterogeneity of outcomes. There was evidence that intervention effectiveness was modified by participants’ social characteristics. Minority ethnic groups were found to benefit more from interventions than majority ethnic groups. Single studies with variable quality showed that those with higher education, who were employed, and adolescents with divorced parents benefited more from interventions. The evidence for differences by age, gender, and health literacy was conflicting (eg, in some instances, older people benefited more, and in others, younger people benefited more). There was no evidence of differences in income, numeracy, or household size.

**Conclusions:**

There was evidence that web-based self-care interventions for chronic conditions can be advantageous for some social groups (ie, minority ethnic groups, adolescents with divorced parents) and disadvantageous for other (ie, low education, unemployed) social groups who have historically experienced health inequity. However, these findings should be treated with caution as most of the evidence came from a small number of low-quality studies. The findings for gender and health literacy were mixed across studies on diabetes, and the findings for age were mixed across studies on asthma, COPD, and diabetes. There was no evidence that income, numeracy, or the number of people living in the household modified intervention effectiveness. We conclude that there appear to be interaction effects, which warrant exploration in future research, and recommend a priori consideration of the predicted interaction effects.

**Trial Registration:**

PROSPERO CRD42017056163; https://www.crd.york.ac.uk/prospero/display_record.php?RecordID=56163

## Introduction

### Chronic Illness and Health Inequalities

Chronic or long-term conditions such as diabetes have a significant impact on the individual’s quality of life and are the major cause of disability and premature death worldwide [1,2]. In high-income countries, chronic conditions are estimated to account for 87% of all deaths [3]. Due to their nature, they cause illness over long periods and their management is complex and costly [2]. In countries where health care is universally provided (such as the National Health Service [NHS] in the United Kingdom), services are struggling with the increasing demand, partly driven by the increasing number of people with chronic conditions [4-7]. Even in high-income countries, people living in constrained conditions and with a lower socioeconomic status (SES) experience chronic illness more commonly and severity is greater than average [2,8]. For example, 52% of those from unskilled occupations suffer from chronic conditions in comparison with 33% of those in professional occupations [9]. These inequalities in health have been attributed to social determinants of health (SDH) and inequity in access to health care [10]. SDH are the complex interacting elements in the physical and social environment that contribute toward disparities in health status. Inequalities in the distribution of good quality health care mean that people do not have equal access to treatments that can improve health outcomes. Taken together, SDH mean disadvantaged groups suffer more illness and more severe illness, but are least likely to receive effective treatment, which together result in disparities in health outcomes [11].

### Proposed Solutions to Increase Access to Health Care for People With Chronic Conditions

Both self-care and web-based interventions have been proposed as methods for increasing access to health care while relieving pressure on health care services. The underlying assumption with self-care interventions is that they provide health care where there was none, by encouraging people to be their own health resource [12]. Web-based interventions have the potential to increase access to good quality health care by providing support to an almost unlimited number of people from the same digital platform at the same time, and the interventions can be tailored to individual needs [13-16]. More recently, combinations of the 2 approaches, in the form of web-based self-care interventions, have become more prevalent for a range of health conditions, particularly for chronic conditions [17,18]. These digital self-care interventions are viewed as playing a vital role in the prevention and treatment of long-term illnesses such as chronic lung disease, cancer, cardiovascular disease, and diabetes [18,19].

### The Impact of Electronic Health and Self-Care Interventions on Health Inequalities

Evidence of the impact of these approaches on health inequalities has been mixed. Nondigital self-care interventions in the form of community-based training courses have been found to improve health status, health behaviors, and the quality of life of patients with chronic conditions [20-24]. However, there is evidence that disadvantaged groups face barriers to accessing these interventions, such as the high levels of health literacy that are often required to understand the training materials as well as language barriers where the training is only conducted in English [8]. Web-based interventions designed specifically for those from underserved and disadvantaged groups have been found to benefit these populations [14,25,26]. However, there is also evidence that access and usability for disadvantaged groups remain to be barriers [27-30]. People from lower socioeconomic groups and older adults are less likely to seek health information on the web and have problems using the web-based information available [31-38]. There is an absence of systematic review evidence investigating whether web-based self-care interventions designed for people with chronic conditions are equally effective for people with different social characteristics.

## Methods

A systematic review was conducted following the Cochrane review guidelines [39] and was reported using the Preferred Reporting Items for Systematic Reviews and Meta-Analyses-Equity 2012 extension checklist [40]. The protocol was registered in advance on the PROSPERO international prospective register of systematic reviews (registration number CRD42017056163).

### Objective

This study aimed to synthesize evidence investigating whether web-based self-care interventions designed for people with chronic conditions are equally effective for people with different social characteristics.

### Inclusion Criteria

#### Population

A total of 4 high burden physical health conditions were included: asthma, diabetes mellitus (type 1 and type 2), osteoarthritis, and chronic obstructive pulmonary disease (COPD). The health conditions were identified using the World Health Organization’s disease burden data and were selected from the top 10 conditions that cause the greatest number of years lost to disease [2,41-44]. All included conditions cause considerable burden and disability to patients [41] and health services [42,43] and have been shown to have social patterning in severity and incidence [2,44]. Furthermore, all 4 have the potential for symptoms, severity, and prognosis to be improved through changes to behavior, such as diet or physical activity.

#### Intervention

We included interventions that were aimed at improving symptoms or prognosis and had a web-based component or were delivered exclusively on the web. This included mobile apps with web connectivity. Interventions were included if they were predominantly reliant on the individual changing their self-care behavior without intensive contact with a therapist or clinician.

#### Study Types

Studies with a quantitative design were included, such as randomized controlled trials (RCTs), observational studies, quasi-experimental designs, feasibility and pilot studies, and mixed methods studies that included a quantitative element. Abstracts were not included where no full publication or report was available [45].

#### Systematic Review Outcomes

Studies reporting health, behavior, knowledge, and psychosocial outcomes were included.

#### Available Data

Studies were included if the study teams had explored whether the social or cultural groups had modified intervention effectiveness and whether the independent contribution of the group on the outcome could be determined. The authors were contacted for models where the independent contribution of the social group could not be determined in the text.

### Exclusion Criteria

There were no language restrictions. The publication dates were limited from January 1, 2006, to February 20, 2019, to ensure that the review included interventions with recent technology.

#### Search Strategy

We conducted searches in Ovid Medical Literature Analysis and Retrieval System Online (MEDLINE; Allied and Complimentary Medicine [AMED], Excerpta Medica dataBASE [EMBASE], and PsycINFO) and Cumulative Index to Nursing and Allied Health Literature (CINAHL) databases. The final search strategy included terms for web, health conditions, self-care, or behavior change. The final search strategies are available in the Multimedia Appendix 1. Corresponding authors were contacted for additional publications, including where only abstracts were located in the search. References of the included papers were screened for inclusion.

#### Study Selection

After deduplication, screening was performed in 2 stages. In stage 1, abstracts and titles were screened. In line with previous practice where a large number of studies were located, partial double screening with checks for accuracy were used [46]. A random 10% sample of the abstracts and titles were independently double screened for inclusion by ST and a single second researcher [47]. Agreement between reviewers of the 10% of titles and abstracts was good (87.5%, where the prevalence-adjusted and bias-adjusted kappa=0.75 indicates good agreement) [48]. Disagreement was resolved by discussion, and consensus and screening tools were refined in light of these. The remainder of the title and abstracts were reviewed by ST only. At stage 2, full texts were obtained and screened for inclusion by ST [49,50].

### Data Extraction

One author (ST) extracted the data from the included studies. Where more than one outcome was reported in each category, the primary outcome was included. Quality of life (QoL) was categorized as a health or psychosocial outcome depending on whether there was a greater balance of health or disability or psychosocial questions in the QoL tool.

The PROGRESS Plus (PP) framework was used to identify the SDH that could contribute toward health inequalities in the included health conditions and in the context of web-based interventions [51]. The analyses that had explored the modification of intervention effectiveness by social characteristics in the PP framework were extracted.

### Risk of Bias (Quality) Assessment

Methodological quality was assessed independently by ST and either PL, CC, or AH. Disagreement was resolved by discussion, and consensus was reached for all of the risk of bias (RoB) domains. The Cochrane collaboration RoB tool 1.0 was used to assess the quality of RCT studies [52]. The newly updated version 2.0 was not used as it does not allow for assessment of *other bias* and would therefore not allow us to capture issues with selective recruitment. The Risk Of Bias In Non-randomized Studies-of Interventions (ROBINS-I) tool was used to assess the quality of non-RCT studies [53]. These tools were used to produce an overall RoB rating for each study.

Much of the Cochrane RoB assessment is focused on ensuring that there is balance in the samples in the two arms of the study. However, the potential for selective recruitment is also important to ensure that the sample is representative of the population with the condition. Here, selection bias was assessed under the *other* category. Inclusion and exclusion criteria were examined to determine whether they potentially excluded people who experienced greater health inequity (eg, no access to the internet, not having the skills to use it, language barriers) and whether there was a discussion of the study population being representative of those with the condition.

### Data Analysis and Synthesis

Descriptive tables were populated using the data from the included papers, accompanied by a narrative synthesis. A meta-analysis was not possible due to the heterogeneity of outcomes. A novel summary figure was developed for this systematic review, which was based on an adapted version of the Harvest plot, referred to here as the *Adapted Harvest plot* [54]. The Adapted Harvest plot allows for a direct comparison of the sample size of the studies where the effect was found (or not found) across outcomes, and an impression of the quality of the study through RoB. This gives an indication of the strength and validity of the findings in relation to each outcome. A key explanation of the features and representation of the Adapted Harvest plot is shown in Multimedia Appendix 2, and information is provided in each plot. To be inclusive, all reported trends (*P*<.10) were included as evidence regardless of whether they fell under the standard *P*<.05 probability cut off and were reported in the text.

### Analysis of Subgroups or Subsets

We intended to conduct subgroup analysis of differences in the application of behavioral change techniques and theory in intervention development and differences in use as potential mechanisms for modification of intervention effect by the PP group. However, these were inconsistently reported, making it challenging to analyze and draw any conclusions. Only 7 of the studies reported modification of use by PP characteristics, and 11 described the use of theory in intervention development. The selection and application of behavioral change techniques have largely not been described. Therefore, the exploration of mechanisms for modification of the intervention effect did not progress to a full analysis.

## Results

### Selection of Studies

**Figure 1:**
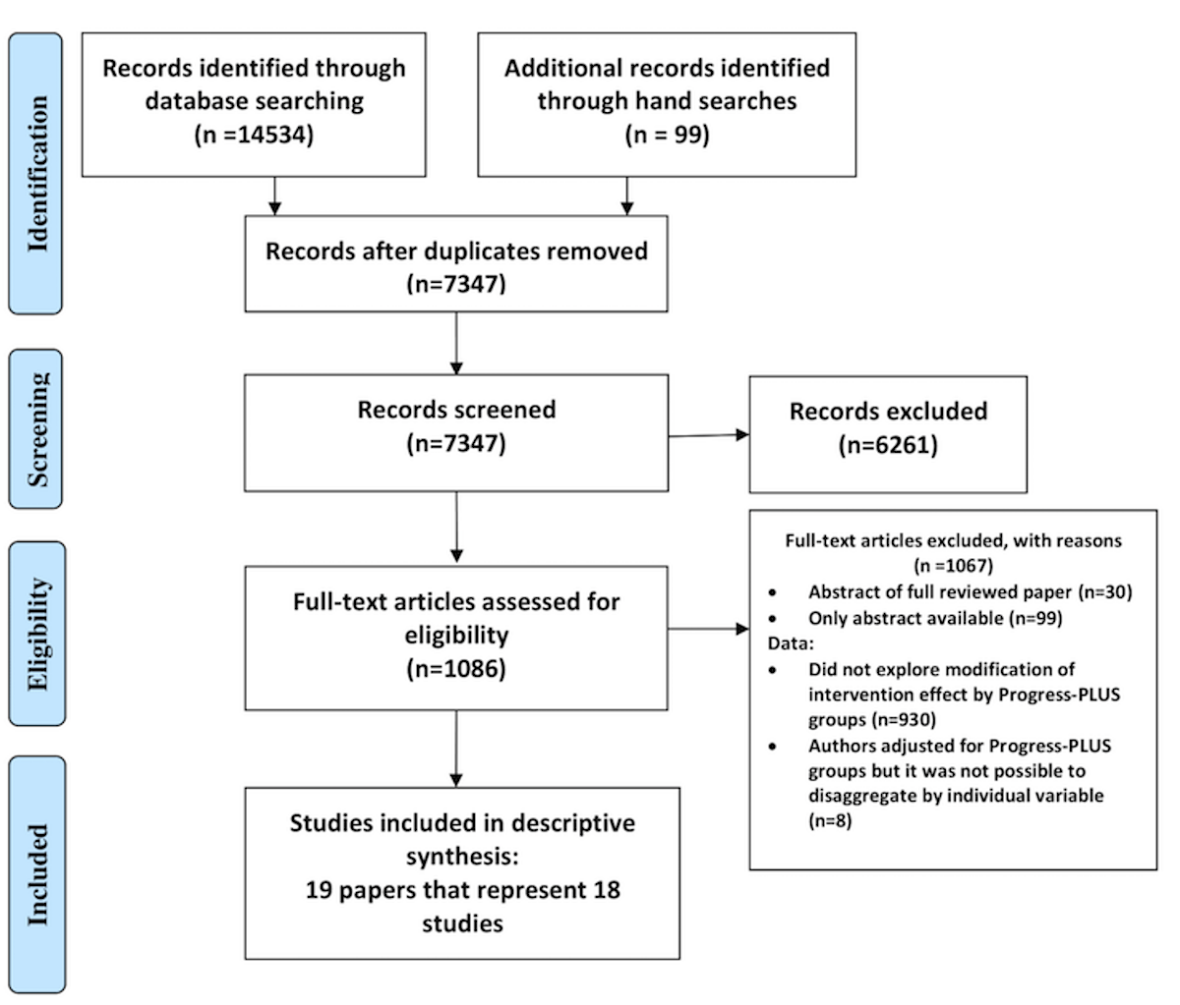
Preferred Reporting Items for Systematic Reviews and Meta-Analysis flow chart.

After the removal of duplicates, 7346 records were obtained (Figure 1). A total of 18 studies (reported across 19 papers) were eligible for inclusion in the review (Figure 1).

### Description of Studies

Of the 18 included studies, 1 focused on asthma [55], 2 on COPD [56,57], 13 on diabetes (5 on type 1 [58-62], 6 on type 2 [63-68], and 2 on both [69,70]), and 2 studies included participants with osteoarthritis [71,72]. The study characteristics are described in Multimedia Appendix 2. In total, 9 of the studies were conducted in the United States, 3 in the Netherlands, 2 in Canada, and 1 in the United Kingdom, Australia, France, and Israel; 13 of the studies were RCTs and 5 were non-RCTs. In total, 6003 participants were included, and the study size ranged from 38 to 1799. The reported characteristics of the study participants are presented in Multimedia Appendix 3. Studies of both children and adults were included, with an age range of 12 to 75 years across the studies. Half of the participants were female (50.1%).

Only 1 study reported that the patients enrolled in the study were representative of the population with diabetes [69]. In total, 4 studies purposely recruited a highly diverse sample in terms of ethnicity [62,67,69,73]. Follow-up times varied from 2 weeks to 12 months, and the follow-up time point was not clear for 1 study [69]. Study attrition ranged from 0% [67,70] to 31% [65], and attrition was not clearly stated in 2 studies [69,71].

### Intervention Content and Outcomes Targeted by the Intervention

In total, 4 of the studies explored the effectiveness of smartphone apps and the remaining 14 explored interventions delivered through websites. Descriptions of the intervention content are provided in Multimedia Appendix 4 and summarized below. In addition to the website or app, 3 interventions provided remote support by phone or video call [55,63,65,72], 1 provided a workbook [67], 1 provided a blood glucose monitor with wireless transfer [69], and 1 provided motivational interviewing before use of the website [61]. In 3 studies, there were 2 versions of the intervention, 1 with and 1 without external support (eg, email or phone support) [60,64,67].

#### Modification of Intervention Design for the Needs of Disadvantaged Social Groups

A total of 2 studies designed the intervention so it had an accessible format: one was designed to maximize usability for people with lower health literacy [65] and the other study used a serious-game intervention designed to be appealing to a range of ages (11-18 years) and a range of baseline knowledge levels in boys and girls [59]. Studies also designed interventions so they were appealing or accessible to ethnic minority groups. TEENCOPE was developed specifically for young people and used a graphic novel format and a cast of ethnically diverse characters with type 1 diabetes (T1D) who present challenging social situations, approaches to solving problems, and consequences of decisions [62]. Another study that targeted Latinos for a type 2 diabetes (T2D) self-management program was provided in Spanish and English [64,67,73].

#### Overall Duration of Intervention

Intervention duration varied from 4 weeks to 12 months, and 5 studies did not provide clear information on the duration [60,64,68,69,71]. 

#### Potential for Meta-Analysis

It was not possible to conduct meta-analysis due to differences in the outcomes and PP categories included in the modification of intervention effect analysis, resulting in a high level of heterogeneity. Narrative synthesis was used to present findings in relation to the research questions for each of the 4 health conditions.

#### Methodological Quality

##### Randomized Controlled Trial

**Figure 2:**
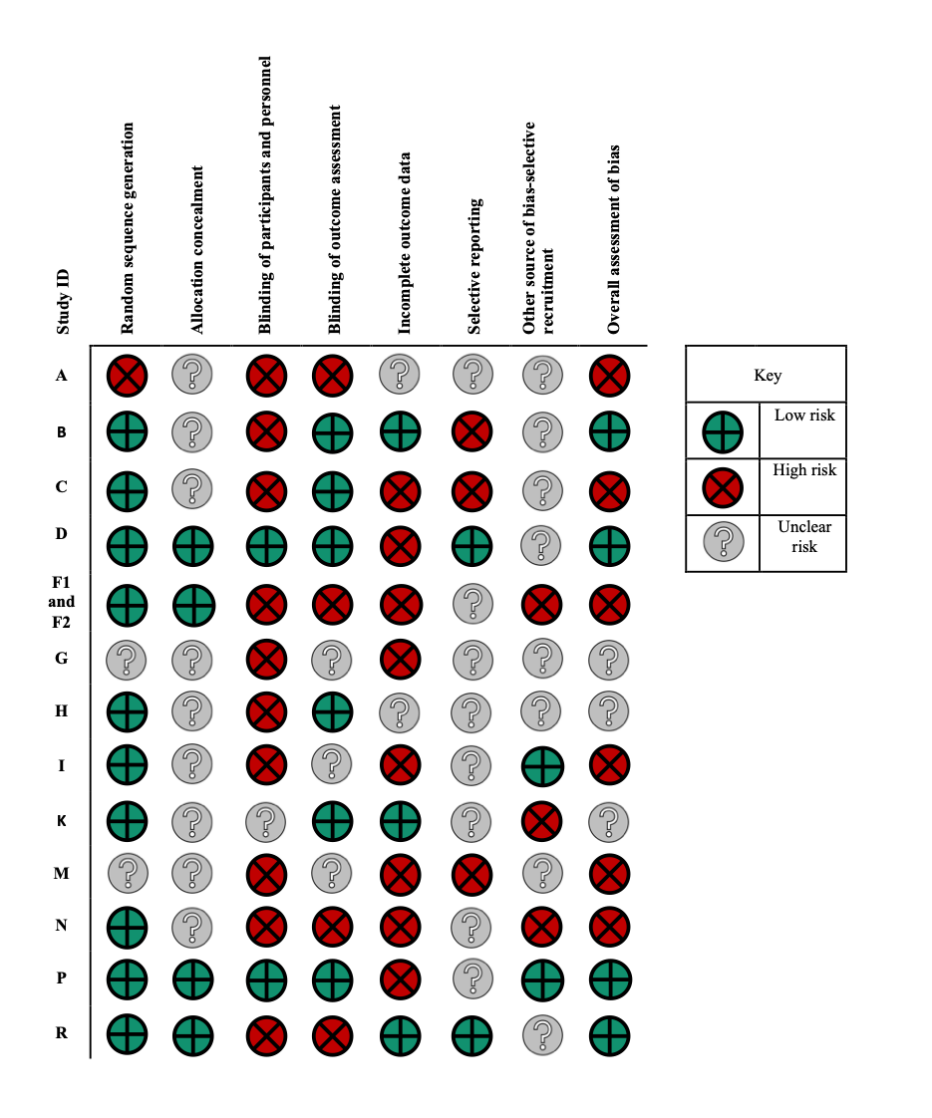
Risk of bias table for randomized controlled trials.

Using the Cochrane RoB assessment, 4 of the 13 RCTs were considered to be low-risk RoB [56,62,63,72], 6 had high RoB [55,57,60,61,64,69], and 3 did not have enough information to assess RoB [58,66,67]. Methodological assessments for each of the domains in the Cochrane RoB assessment are provided in Figure 2. Overall, the lowest RoB came from the random sequence generation (low RoB in 10 studies, high in 1, and insufficient information was provided in 2 studies) and the highest came from blinding of participants and personnel (10 studies had high RoB, 2 had low, and 1 was unclear). However, given the nature of the digital interventions, it was often not possible to blind the participants and personnel. Selection bias was assessed under the *other* category, with 8 studies classified as unclear RoB, 3 as high, and 2 as low RoB.

##### Nonrandomized Controlled Trial Studies

**Figure 3:**
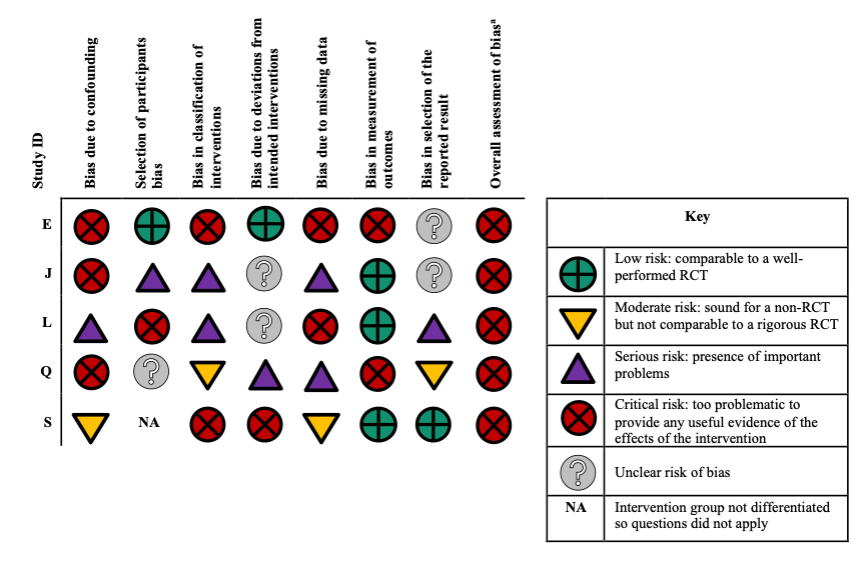
Risk of bias assessment for the nonrandomized controlled trial (RCT) studies. ^a^Overall risk of bias: equal to the most severe level of bias found in any domain.

Using ROBINS-I, all 5 of the non-RCT studies were considered to have critical RoB (Figure 3) [59,65,68,70,71]. Across the studies, the lowest RoB came from the measurement of outcomes, and the highest RoB came from the classification of the intervention, and bias due to missing data.

### Modification of Intervention Effectiveness by PROGRESS Plus Groups

A total of 15 of the 18 studies that explored the modification of intervention effectiveness by PP categories reported effect modifiers. There was evidence that people were more likely to benefit from the intervention if they were from a minority ethnic group, were employed, had a higher level of education, and had divorced parents (study of adolescents). The findings for age, gender, and health literacy were mixed. There was no evidence of an interaction income, numeracy, or the number of people living in the household. The full key to the Adapted Harvest plot is shown in Table 1. Further details including outcomes and estimates (where provided) where interactions were found are presented in Multimedia Appendix 5 and a matrix table containing an overview of interactions across the conditions and PP groups is provided in in the Multimedia Appendix 6.

**Table 1:** Key to the Adapted Harvest plot.

Feature	Representation
Direction of effect category	Positive: favors the PROGRESS Plus group (*P*<.10) No effect: study found no evidence of an effect (*P*≥.10) Negative: favors the comparator group (*P*>.10)
Stack height	Study size
Stack color	Risk of bias assessment RCT^a^ studies: Low risk—blue, high risk—purple, unclear—gray Non-RCT studies: low risk—blue, moderate risk—yellow, serious—orange, critical—purple, not enough information—gray
Stack pattern	RCT studies: solid colors Non-RCTs: patterned with dots
Number within the stack	Study ID
Bar size	Total number of participants in the studies finding evidence of a positive association, no effect, or a negative association with the outcome

^a^RCT: randomized controlled trial.

#### Age

A total of 14 studies (1 asthma, 2 COPD, 9 diabetes, and 2 osteoarthritis) examined the modifying effect of age on at least one outcome, with evidence of mixed effects (younger and older benefited more) across asthma, COPD, and diabetes studies.

##### Asthma

The one asthma study (ID A; n=234; high RoB) found that increasing age was associated with increased medication adherence among adolescents aged 12 to 18 years [55]. Those scoring in the higher range in the Medication Adherence Report Scale (>19) were on average aged 0.7 years (*P*=.02) than those who scored in the lower range.

##### Chronic Obstructive Pulmonary Disease

**Figure 4:**
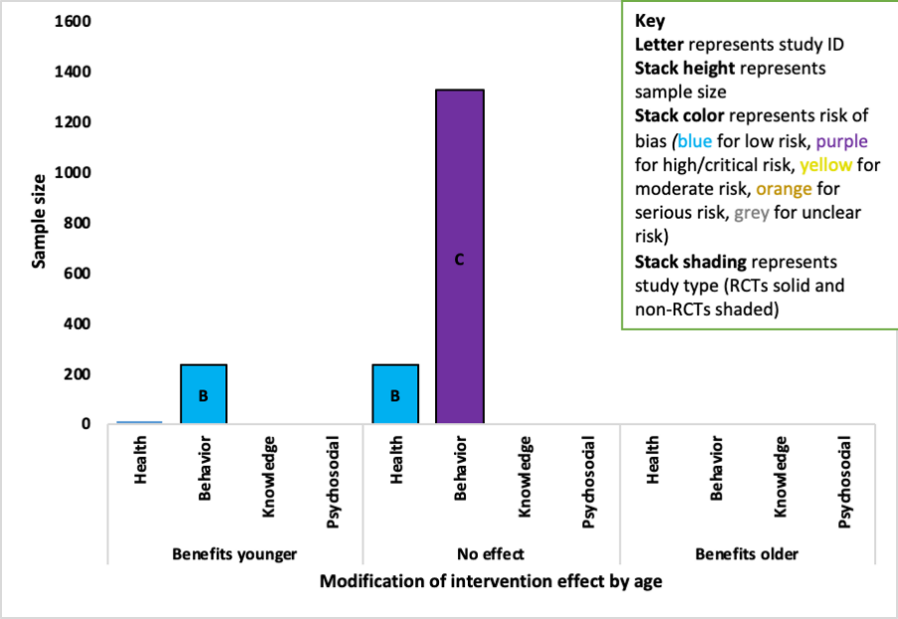
Adapted Harvest plot with evidence for the modification of intervention effect by increasing age for chronic obstructive pulmonary disorder studies. RCT: randomized controlled trial.

One of the 2 COPD studies found evidence that older participants benefited less from the intervention than younger people on the behavioral outcome (Figure 4). The higher quality evidence came from an RCT (ID B; n=239; low RoB) that indicated that a 1-year increase in age was associated with a 33-point decrease in change in daily step count (*P*=.03) but found no association with the health-related QoL outcome [56]. The evidence for no effect on the behavioral outcome came from an RCT (ID C) with a high RoB but a larger sample (n=1325) [57].

##### Diabetes

**Figure 5:**
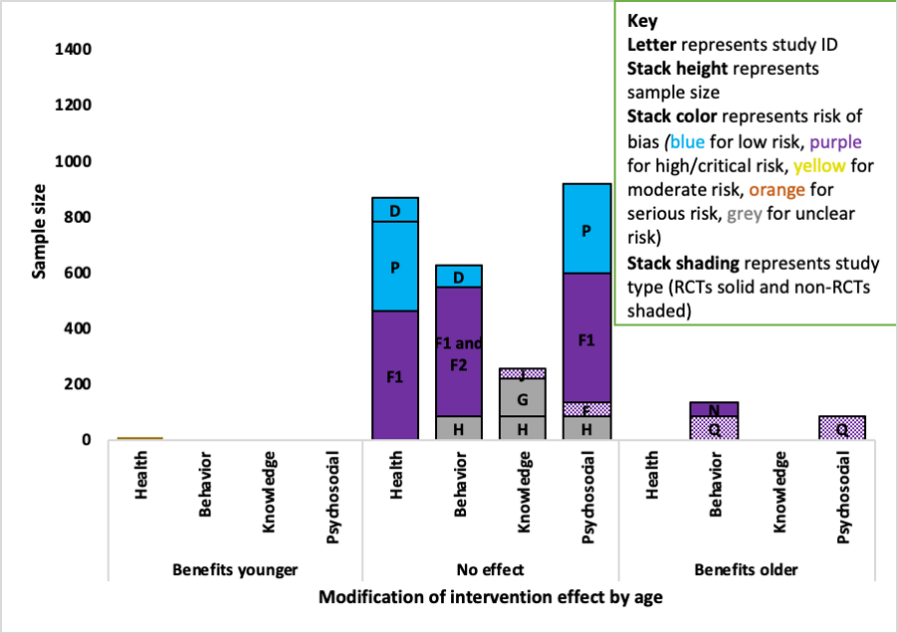
Adapted Harvest plot with evidence for increasing age-modifying intervention effects across outcomes in diabetes studies. RCT: randomized controlled trial.

Of the 9 diabetes studies that reported the modification of intervention effectiveness by age, 2 studies indicated that older participants benefited more from using the intervention (Figure 5).

For the behavioral outcome, the strongest evidence indicated that there was no modification of intervention effect by age, which came from 3 studies (IDs D, F, and H) with an overall sample of 628 patients and a low, high, and unclear RoB [58,63,64,73]. There was evidence that older participants benefited more in 2 small studies (combined n=133) with a high and critical RoB: a non-RCT (ID Q; n=81; critical RoB) found that older participants benefited more (*P*<.001) in adults aged ≥25 years with T2D [68], and an RCT (ID N; n=52; high RoB) found older adolescents (aged 16-18 years) improved more (*P*<.01) than younger adolescents with T1D (aged 13-18 years) [61].Regarding psychosocial outcomes, the strongest evidence indicated that there was no modification of effect by age and it came from 4 studies (IDs P, F, E, and H, combined sample n=915) with low, unclear, high, and critical RoB [58,62,64,65]. Whereas evidence for modification for intervention effect on the behavioral outcome (*P*=.01) in adults aged ≥25 years with T2D came from a small non-RCT (ID Q; n=81; critical RoB) [68]. There was no evidence of an interaction effect with age across the health outcomes in 3 studies (IDs D, P, and F) [62-64] or with diabetes knowledge in 3 studies (IDs J, G, and H) [58,59,66].

##### Osteoarthritis

There was no evidence of a difference in effectiveness by age for health outcomes in the 2 studies [71,72].

#### Gender

A total of 12 studies (8 diabetes, 1 asthma, 1 COPD, 2 osteoarthritis) examined the modifying effect of sex on at least one outcome, with mixed findings in diabetes studies.

##### Asthma, Chronic Obstructive Pulmonary Disease, and Osteoarthritis

There was no evidence of a difference in effectiveness by gender on a behavioral outcome (medication adherence) in the asthma study (ID A) [55], a behavioral outcome in the COPD study (ID C) [57], or on health outcomes in either of the osteoarthritis studies (IDs R and S) [71,72].

##### Diabetes

**Figure 6:**
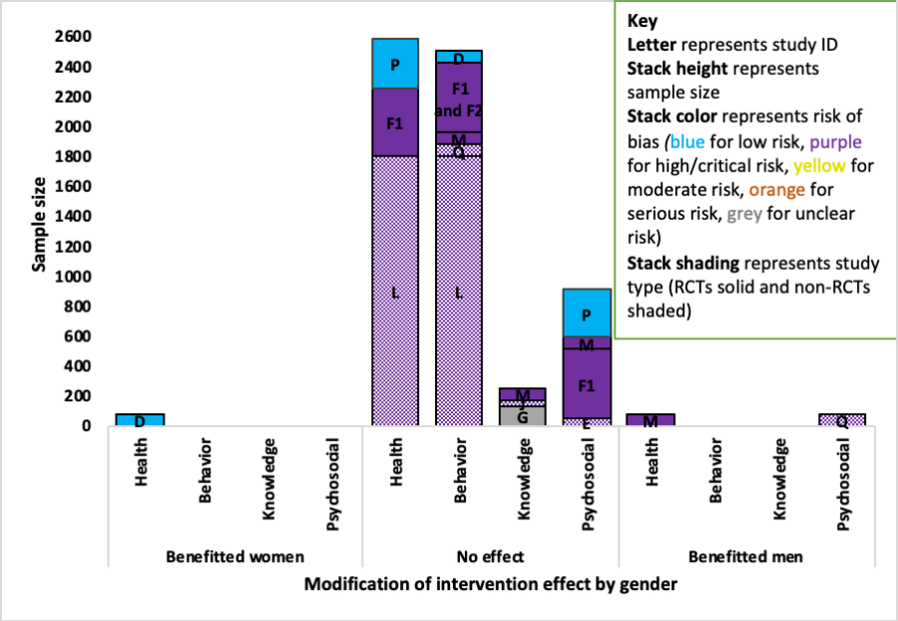
Adapted Harvest plot for gender-modifying intervention effect across outcomes in diabetes studies. RCT: randomized controlled trial.

Of the 9 diabetes studies that explored the modification of intervention effect by gender, 3 small studies (IDs D, M, and Q) found evidence of a difference. Two studies (IDs M and Q) indicated that male participants benefited more, and one study (ID D) indicated that female participants benefited more (Figure 6).

The evidence that gender modified the health outcome was mixed. The strongest evidence indicated that gender did not modify the intervention effect and it came from 3 studies (IDs P, F1, and L; combined n=2582) with low, high, and critical RoB [62,64,70]. Of the studies that indicated gender-modified intervention effectiveness, one small RCT (ID D; n=84) with a low RoB indicated female participants benefited more (*P*=.03) [63] and a small RCT (ID M; n=79) with a high RoB indicated male participants benefited more (*P*=.06) [60].

For psychosocial outcomes, all of the evidence came from studies of high and critical RoB. The evidence that male participants benefited more (*P*=.01) on psychosocial outcomes came from one small non-RCT (ID Q; n=81) with a high RoB [68], in comparison with 4 studies (IDs P, M, F1, and E) that found no evidence of an effect with a combined sample of 862 and low, high, critical, or unclear RoB (Figure 6) [60,64,65]. There was no evidence of the intervention effect being modified by gender for the behavioral outcomes in 5 studies (IDs D, F, M, Q, and L) or knowledge outcomes in 3 studies (IDs M, J, and G) [60,64,66,68,73].

#### Education

A total of 7 studies (5 diabetes, 1 COPD, and 1 osteoarthritis) examined the modifying effect of education as an outcome, with evidence that higher education benefited more in 1 diabetes study.

##### Chronic Obstructive Pulmonary Disease and Osteoarthritis

There was no evidence that the levels of education-modified intervention effectiveness in one COPD (ID C) study on a behavioral outcome [57], or an osteoarthritis study (ID R) on health outcomes [72].

##### Diabetes

**Figure 7:**
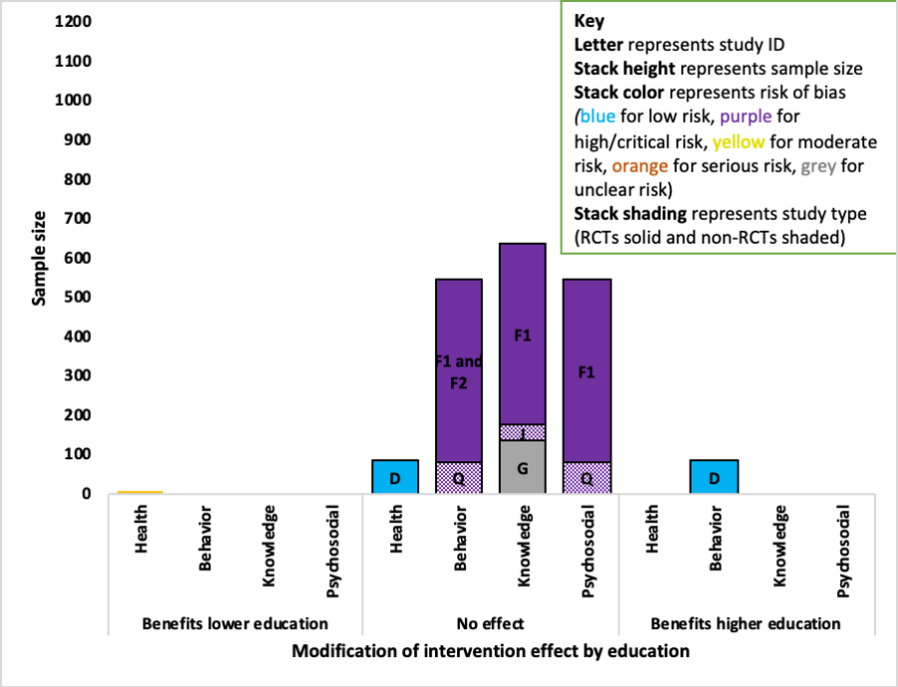
Adapted Harvest plot for higher education modifying intervention effects across outcomes in diabetes studies. RCT: randomized controlled trial.

There was evidence that those with higher education benefited more in one study, and no evidence of a difference in 4 diabetes studies (Figure 7). The strongest evidence came from a small RCT (ID D; n=84) with low RoB, which indicated that those with higher education benefited more from the intervention on a behavioral outcome (*P*=.03) but did not find an effect on a heath outcome [63]. The combined sample of the 2 studies where there was no effect on the behavioral outcome was 544, with a high (ID F1 and F2; n=463) [64,73] and critical RoB (ID Q; n=81) [68]. There was no evidence that education-modified intervention effect on knowledge outcomes in 3 studies (IDs F1, J, and G) [59,64,66] or psychosocial outcomes in 2 studies (IDs F1 and Q) [64,68].

#### Ethnicity

A total of 8 studies (7 diabetes and 1 osteoarthritis) examined the modifying effect of ethnicity on study outcomes, with evidence that ethnic minority groups benefited more from the interventions in diabetes studies.

##### Diabetes

**Figure 8:**
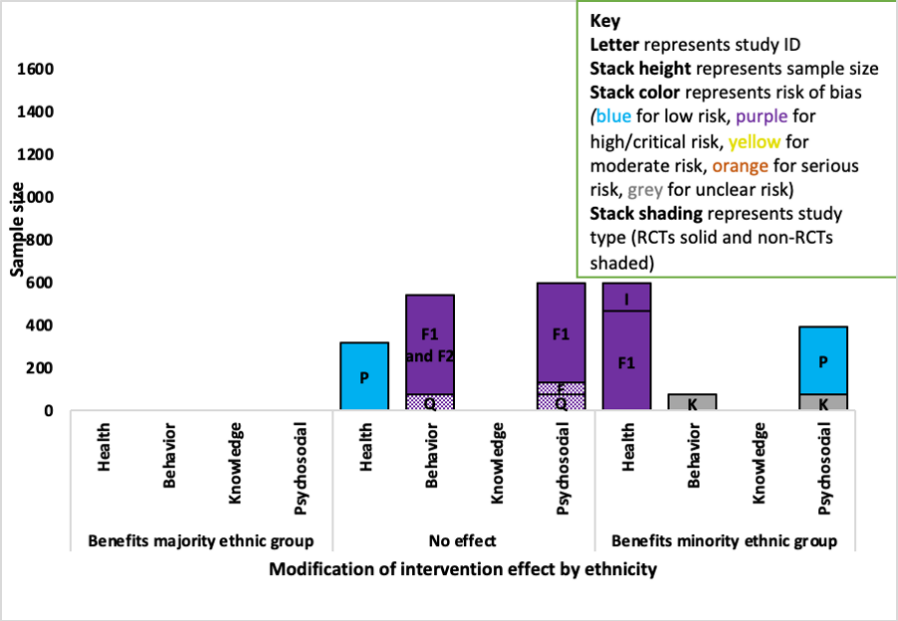
Adapted Harvest plot for minority ethnic group modifying intervention effects across outcomes in diabetes studies. RCT: randomized controlled trial.

There was evidence that minority ethnic groups benefited more from the intervention than majority ethnic groups in 4 of the 7 diabetes studies that explored this interaction (Figure 8).

For the health outcomes, an RCT study with a low RoB (ID P; n=320) found no evidence that the intervention effect was modified by ethnicity [62], while 2 RCTs (ID F1 and I; combined sample n=597) both with a high RoB indicated that people from minority ethnic groups benefited more on the health outcome (ID F1; *P*=.006; for study ID I no estimates were provided) [64,69].

Regarding the behavioral outcome, a small RCT (ID K; n=73) with an unclear RoB found that minority ethnic groups benefited more (*P*=.01) [67], and the evidence of no effect came from one non-RCT (ID Q; critical RoB) and an RCT (ID F; high RoB) with a combined sample of 544 [64,68,73].

For the psychosocial outcomes, evidence indicating that minority groups benefited more from the intervention came from 2 RCTs (combined sample n=393): ID P with a low RoB (n=320; *P*=.07) [62] and ID K (n=73; *P*=.003) with an unclear RoB [67]. The evidence of no effect on the psychosocial outcome came from 3 studies (IDs F1, E, and Q; combined sample n=595): an RCT with a high RoB and 2 non-RCTs with critical RoB [64,65,68]. No studies reported the modification of a knowledge outcome by ethnicity.

##### Osteoarthritis

There was no evidence that ethnicity-modified intervention effect on a health outcome in adults with osteoarthritis in a non-RCT (ID S) [71].

#### Employment

A total of 3 studies (1 COPD, 1 diabetes, and 1 osteoarthritis) examined the modifying effect of employment on at least one outcome, with evidence that employed participants benefited more in an osteoarthritis study.

##### Chronic Obstructive Pulmonary Disease and Diabetes

There was no evidence that employment was a moderator of intervention effectiveness in one RCT COPD study (ID C) [57] or one non-RCT diabetes study (ID Q) [68].

##### Osteoarthritis

An osteoarthritis RCT with a low RoB (ID R; n=148) found that participants who were employed showed greater improvements in health outcomes (walking pain) than unemployed participants 3 months after using the intervention (interaction: *P*=.02) [72].

#### Health Literacy

**Figure 9:**
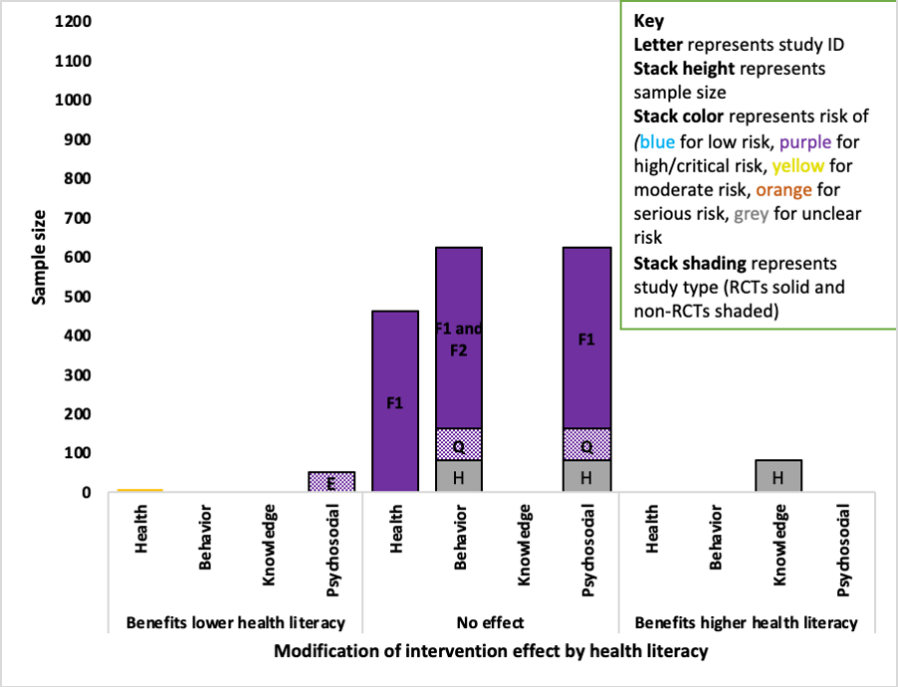
Adapted Harvest plot for higher health literacy modifying intervention effects across outcomes in diabetes studies. RCT: randomized controlled trial.

A total of 4 diabetes studies examined the modifying effect of health literacy on study outcomes, with 2 of the 4 diabetes studies reporting evidence of a difference. However, the 2 studies provided evidence in different directions for different outcomes (Figure 9). The evidence from both studies was weak: a small RCT (ID H; n=81) with an unclear RoB found evidence that people with higher health literacy benefited more from the intervention on a knowledge outcome (no estimates provided) [58].

For psychosocial outcomes, 3 studies (combined sample: n=625) indicated that health literacy did not modify the intervention effect; these were 2 RCTs with an unclear (ID H) and high RoB (ID F1) and a non-RCT with a critical RoB (ID Q) [58,64,68]. A small single-arm pilot non-RCT (ID E; n=51) with a critical RoB found that those with lower health literacy benefited more on psychosocial outcomes (*P*=.02) [65]. There was no evidence that health literacy modified the intervention effect on a health outcome in 1 study (ID F1) [64] or behavior change outcomes in 3 studies (IDs F, Q, and H) [58,64,68,73].

#### Family Structure

One small non-RCT (ID J; n=38) diabetes study with a critical RoB examined the modifying effect of family structure on study outcomes, finding children of divorced parents benefited more from the intervention. A higher proportion of children (aged 11-18 years) with T1D who were in the high-effect subgroup for change in diabetes knowledge (benefited more from the intervention) had divorced parents (35%) compared with the low-effect subgroup (6%; *P*=.03) [59].

#### Income, Numeracy, Number of People Living in the Household

There was no evidence that income modified effectiveness on 2 diabetes studies (1 non-RCT: ID Q and 1 RCT: ID P) [62,68] or numeracy in 1 RCT (ID F) [64]. There was no evidence that the number of people living in the household modified the effectiveness of an intervention on health outcomes in a non-RCT osteoarthritis study (ID S) [71].

## Discussion

### Principal Findings

This review examined the modifying effects of participant characteristics on the effectiveness of web-based interventions for chronic health conditions. We found evidence that intervention effectiveness was modified by participants’ social characteristics. In the diabetes literature, there was evidence that people from minority ethnic groups gained greater benefits from interventions than majority ethnic groups. There was evidence from single studies with variable quality that those with higher education, divorced parents (adolescent), and who were employed benefited more from interventions. A small high-quality diabetes study indicated that those with a higher level of education benefited more from the intervention. A small low-quality diabetes study found that a higher proportion of adolescents with divorced parents (adolescents) were in the high-effect intervention group compared with the low-effect group. A high-quality osteoarthritis study indicated that employed participants were more likely to benefit from the intervention than unemployed participants. The findings for modification of the effect by participant age were mixed. Older people were found to benefit less from the intervention in high-quality COPD studies, and they were found to benefit more from the intervention in 2 low-quality diabetes studies and a low-quality asthma study. Gender and health literacy were only considered in studies on diabetes and showed mixed effects. A total of 2 small low-quality studies indicated that male participants benefited more from diabetes interventions, and 1 small high-quality study indicated that female participants benefited more. Two studies had contradicting findings for health literacy: a small study with an unclear RoB indicated that those with higher health literacy benefited more, while a small low-quality study found that those with lower health literacy benefited more. There was no evidence of a modification to intervention effectiveness by income, numeracy, or the number of people living in the household.

The strength of evidence across the studies could not be assessed through a meta-analysis as not all studies provided estimates, and the outcomes were heterogeneous. Therefore, the strength of the evidence was explored through study size, RoB, and estimates, where possible. The majority of the evidence was drawn from studies with high and uncertain RoBs.

### Strengths and Limitations of the Methods Used

To our knowledge, this is the only systematic review that has investigated whether there are equal benefits of web-based self-care interventions for people with different characteristics. The breadth of the studies reviewed here is both a strength and a weakness. The inclusion of 4 different physical health conditions meant that it was not possible to combine the evidence in cases where the outcomes targeted were heterogeneous, which limits what can be inferred from the results. However, we summarized the available evidence, providing the first systematic exploration of how PP characteristics modify intervention effectiveness. Simple vote counting was not judged appropriate for the synthesis of findings that could not be meta-analyzed. Instead, we created a novel summary figure based on the Harvest plot referred to here as the *Adapted Harvest plot*. The Adapted Harvest plot provided an indication of the strength of the evidence for narrative synthesis by including study size and RoB.

The majority of the screening was conducted by one person, and only 10% of the abstracts and titles were double screened. This is in line with previous practice where a large number of studies were located [46,74-77]. Every effort was made to locate all relevant literature; however, it is possible that some relevant studies may have been missed.

### Limitations of the Evidence Base

There were several limitations in the methodology and data reported by the studies included in the review. It was not possible to conduct a meta-analysis because the outcomes were heterogeneous, and essential data were not reported. A high proportion of the included studies found that the intervention effect was modified by at least one participant characteristic on at least one outcome. This suggests that teams may be more likely to publish these analyses when they find evidence of a difference in effect [78].

There was a high risk of selection bias across the included studies, which may have excluded people from lower SES groups. The few studies that did comment on the representativeness of the study population indicated that the samples tended to be more white people, with higher levels of education. This not only limits the generalizability of the study findings but also potentially masks differences in effectiveness that may have been present between more and less advantaged groups because the sample is underpowered to detect differences in these subgroups.

The complex relationship between social characteristics and potential effectiveness or engagement with online interventions was not considered carefully in the included studies. The range of different PP characteristics explored and the comparisons within the PP characteristic appeared to be decided post hoc and did not appear to be guided by theory. Therefore, the evidence on the influence of PP on effectiveness was limited.

The Cochrane RoB 1.0 tool was limited in evaluating bias in the data that were important for this systematic review. Although the tool is very effective at identifying bias that can arise from the arms of a study with an unbalanced sample, it does not provide a category that evaluates the risk of the sample not being representative of the general population with the condition. RoB 2.0 was not used in this study because it does not allow for *other* RoBs and would therefore not allow us to capture issues with selective recruitment.

### Comparison With Prior Work

There is no previous systematic evidence comparing the health equity effects of web-based self-care interventions for people with different social characteristics. This review agrees with previous evidence from single studies that have found that web-based self-care interventions can benefit underserved and disadvantaged groups when the intervention has been designed specifically for such groups [14,25,26]. Some of the included studies that found evidence that underserved groups benefited more from the intervention had modified their interventions to be more accessible, useable, or engaging for these groups. Two studies designed the intervention, so it was in an accessible format for those with different educational abilities. A study found that those with lower health literacy benefited more and designed interventions to maximize usability for people with lower literacy [65]. A study found that those with divorced parents and lower baseline knowledge benefited more from the intervention and opted for a serious-game intervention that was designed to be appealing to a range of ages (11-18 years) and therefore a range of baseline knowledge in boys and girls [59].

Of the 4 studies that found that ethnic minority groups benefited more, 2 studies had adapted the intervention to be appealing to the study population targeted. One study targeting Latinos with T2D provided the intervention in Spanish and English [64,73]. Another study that found that ethnic minority youth with T1D benefited more from using the intervention than majority ethnic groups used an intervention with a graphic novel format and a cast of ethnically diverse characters with T1D [62]. However, there are no specific design features that suggest why ethnic minority groups may benefit more than majority ethnic groups in these studies. Indeed, the other 2 studies that found ethnic minority groups benefited more did not adapt the interventions to make it more accessible to ethnic minority groups. It is possible that in these studies, ethnic minority groups may have benefited more from the intervention because they had less exposure to health care support before using the digital intervention. There has been previous evidence that those from ethnic minority groups and those with lower SES face greater challenges accessing health care services and support [79-81]. Therefore, interventions that reduce barriers to access and use may be more effective for populations currently underserved by health care services.

Common to the 4 studies that found minority ethnic groups benefited more from the intervention was their sampling strategy aimed at maximizing the recruitment of minority ethnic groups [62,67,69,73]. Subsequently, all 4 studies had a high representation of people from ethnic minority groups in the sample, resulting in the sample being powered to detect differences in effectiveness by ethnicity. Webb et al [82] similarly found that recruitment sampling was an important predictor of the effectiveness of an intervention. They found that when theory or predictors were used to select recipients for the intervention, the intervention had the greatest improvements in behaviors [82]. The 4 studies in this review cited the potential for digital interventions to increase access to health care in these minority groups, as motivation for their study design, and target the recruitment of minority groups [62,67,69,73]. Therefore, consideration of the sample where the intervention was evaluated appears to be important in addition to considering the needs of the target population. 

Interventions designed without considering the needs of the users can exclude social groups, and this is the likely cause of the difference in effectiveness found by education and employment. Van Dijk’s theory of the digital divide proposed that if the content of the technology only fulfils the needs of the dominant group (eg, high education, employed) or is challenging to use, those users from the less dominant group will benefit less from the use of the technology [83]. This supposition has been supported by findings that the design of web-based health information can limit the usability of digital interventions for people of lower SES [36,37,84,85].

The mixed findings for age, gender, and health literacy may be associated with whether the interventions were designed considering the needs of people with those characteristics. This was illustrated by the findings for health literacy in this review. Davis et al [65] designed their intervention with the needs of low literacy individuals in mind and found that those with lower literacy levels benefited more from the intervention. Huang et al [58] found that those with higher literacy benefited more and acknowledged that they would need to provide additional support for users with lower health literacy levels at baseline. Alternatively, mixed findings may be related to the participant’s other social characteristics. For example, in studies where older people were found to benefit more from the intervention, they may have had a higher level of education and consequently higher digital skills relative to their younger counterparts. There is growing evidence that individual social characteristics do not work in isolation but interact in complex ways that influence health outcomes [86]. As such, conducting an analysis involving the comparison of individual groups may not be sufficient to establish how digital self-care interventions may impact health inequities.

### Conclusions

There was evidence that web-based self-care interventions for chronic conditions can benefit some (minority ethnic groups, divorced parents) and disadvantage other (low education, unemployed) social groups who have historically experienced health inequity. However, these findings should be treated with caution as most of the evidence came from a small number of low-quality studies. The findings for gender and health literacy were mixed across diabetes studies, and the findings for age were mixed across asthma, COPD, and diabetes studies. There was no evidence that income, numeracy, or the number of people living in the household modified intervention effectiveness. We conclude that there appear to be interaction effects that warrant exploration in future research, and a priori consideration of predicted interaction effects is recommended.

## Appendix

Multimedia Appendix 1 Systematic review search strategies.

Multimedia Appendix 2 Table 2. Characteristics of included studies and populations.

Multimedia Appendix 3 Table 3. Participant characteristics.

Multimedia Appendix 4 Table 4. Intervention description.

Multimedia Appendix 5 Table 5: Overview of available data for social characteristics modifying intervention effects.

Multimedia Appendix 6 Table 6. Modification of effectiveness data available for PROGRESS-Plus categories in the included studies.

## Abbreviations

COPD: chronic obstructive pulmonary disease
NHS: National Health Service
NIHR: National Institute for Health Research
PP: PROGRESS Plus
QoL: quality of life
RCT: randomized controlled trial
RoB: risk of bias
ROBINS-I: Risk Of Bias In Non-randomized Studies-of Interventions
SDH: social determinants of health
SES: socioeconomic status
SPCR: School for Primary Care Research
T1D: type 1 diabetes
T2D: type 2 diabetes
