# Supplementary material for: Health Equity in the Effectiveness of Web-Based Health Interventions for the Self-Care of People With Chronic Health Conditions: Systematic Review
Source: J Med Internet Res. 2020 Jun 5;22(6):e17849. doi: 10.2196/17849 (PMC7305554; doi:10.2196/17849)
Supplement: Multimedia Appendix 1 [file jmir_v22i6e17849_app1.docx]

### Systematic Review Search Strategies

**Final Ovid MEDLINE search:**

Search strategy

| **Type** | **Actions** | **Annotations** |
| --- | --- | --- |
| 1 | exp *Self Care/ or self care.ti,ab. or self-care.ti,ab. or self-management.ti,ab. or self management.ti,ab. or self-regulation.ti,ab. or self regulation.ti,ab. or exp *Self Efficacy/ or self-help.tw. or self help.tw. | 243309 |
| 2 | ((behavio?r* adj3 change$) or behavio?r* change technique$).tw. | 163670 |
| 3 | exp *Behavior Therapy/ | 79623 |
| 4 | behavio?ral counselling.ti,ab. | 396 |
| 5 | lifestyle counselling.ti,ab. | 569 |
| 6 | exp *Cognitive Therapy/ | 44487 |
| 7 | or/1-6 | 495926 |
| 8 | *Internet/ or internet$.ti,ab. | 187113 |
| 9 | (web or web-based).ti,ab. or (world wide web or worldwide web or website*).tw. | 292880 |
| 10 | *Telemedicine/ or (mhealth or m-health or "m health").tw. or (telemedicine or Tele-medicine).tw. or cellphone.tw. or exp Cellular phone/ or ((cell$ or mobile$) adj3 phone$).tw. or (smartphone$ or smart-phone$).tw. or (personal$ adj3 digital$).tw. or "mobile health".tw. or (telehealth$ or tele-health$).tw. or (telecare$ or tele-care$).tw. | 107178 |
| 11 | (e-health or ehealth or "e health").tw. | 9715 |
| 12 | computeri?ed.ti,ab. or ((computer-assist* or computer-based) adj6 (therap* or treatment* or education*)).tw. or (interactive or online or on-line or cellular phon* or mobil* phon*).tw. | 688870 |
| 13 | (app$ adj3 (smartphone$ or smart-phone or mobile$ or phone$)).tw. | 22157 |
| 14 | (*technology/ and *inventions/) or technology?based.tw. | 45 |
| 15 | *mobile applications/ or *video games/ | 16586 |
| 16 | *Therapy, Computer-Assisted/ | 7224 |
| 17 | (Software or software design).tw. | 405490 |
| 18 | or/8-17 | 1470733 |
| 19 | asthma$.tw. or exp *Asthma/ | 395462 |
| 20 | Diabetes mellitus.tw. or exp *Diabetes Mellitus/ or exp *Diabetes Complications/ or diabet$.tw,ot. or (IDDM or NIDDM or MODY or T1DM or T2DM or T1D or T2D).tw,ot. or (non insulin$ depend$ or noninsulin$ depend$ or non insulin?depend$ or noninsulin?depend$).tw,ot. or (insulin$ depend$ or insulin?depend$).tw,ot. | 1502227 |
| 21 | exp Diabetes Insipidus/ or diabet$ insipidus.tw,ot. | 24078 |
| 22 | 20 not 21 | 1483566 |
| 23 | Osteoarthr$.tw. or exp *Osteoarthritis/ or arthrosis.tw. | 195167 |
| 24 | Chronic respiratory disease.tw. or exp *Lung Diseases, Obstructive/ or COPD.tw. or exp *Pulmonary Disease, Chronic Obstructive/ or emphysema$.tw. or (chronic$ adj3 bronchiti$).tw. or (obstruct$ adj3 (pulmonary or lung$ or airway$ or airflow$ or bronch$ or respirat$)).tw. or COAD.tw. or COBD.tw. or AECB.tw. or (chronic obstructive pulmonary disease* or chronic obstructive lung disease*).tw. | 412459 |
| 25 | 19 or 22 or 23 or 24 | 2316764 |
| 26 | 7 and 18 and 25 | 5524 |
| 27 | limit 26 to yr="2006 - 2019" | 5141 |
| 28 | remove duplicates from 27 | 3492 |

**CINAHL** **search strategy**

| **#** | **Query** | **Results** |
| --- | --- | --- |
| S20 | S5 AND S12 AND S18 **Limiters** - Published Date: 20060101-20191231 | 2,209 |
| S19 | S5 AND S12 AND S18 | 2,537 |
| S18 | S13 OR S14 OR S15 OR S16 OR S17 | 181,124 |
| S17 | MH "Life Style Changes" OR TX "life style counselling" OR TX "life-style counselling" | 9,497 |
| S16 | TX "behavio#r* counselling" or MH "Cognitive Therapy+" | 19,908 |
| S15 | MH "Behavior Therapy+" OR MH "Behavior Modification" OR (TX behavio#r* N1 TX change?) OR (TX behavio#r* N1 TX "change? technique?") | 30,237 |
| S14 | (MH "Attitude to Illness") OR TI attitude OR AB attitude | 63,515 |
| S13 | MH "Self Care+" OR TX "self care" OR TX "self-care" OR TX "self-management" OR TX "self management" OR MH "Self Regulation" OR TX "self regulation" OR TX "self-regulation" OR MH "Self-Efficacy" OR TX "self-efficacy" OR TX "self efficacy" | 86,449 |
| S12 | S6 OR S7 OR S8 OR S9 OR S10 OR S11 | 398,606 |
| S11 | MH "User-Computer Interface" OR TX "decision aid?" OR TX "Interactive health communicat*" | 9,962 |
| S10 | (MH "Cellular Phone+") OR TX cellphone OR ((TX cell? or TX mobile?) N1 TX phone?) OR ( (TX smartphone? or TX "smart-phone?") or (TX personal* N1 TX digital?) or TX telehealth? or TX tele-health? or TX telecare* or TX "tele-care*" OR TX interactive or TX online or TX "on-line" or TX "cellular phon*" or TX mobil* TX phon* OR TX telemedicine OR TX "Tele-medicine" | 198,174 |
| S9 | (MH "Video Games+") OR (MH "Games") | 7,961 |
| S8 | MH "Therapy, Computer Assisted" OR TX ehealth OR TX e-health OR MH "Decision Making, Computer Assisted+" OR ((TX computer-assist* or TX computer-based OR TX computeri?ed OR TX computer*) N1 (TX therap* or TX treatment* or TX education*)) | 42,700 |
| S7 | MH "Telemedicine" OR MH "Telehealth" OR MH "Mobile Applications" OR TX mhealth OR TX m-health OR MH "Mobile Applications" OR ((TX application or TX app?) N1 (TX smartphone? or TX smart-phone or TX mobile? or TX phone?)) | 20,939 |
| S6 | MH "Internet+" or TX internet* or MH "World Wide Web+" or TX web or MH "Website Development" or MH "World Wide Web Applications+" | 179,397 |
| S5 | S1 OR S2 OR S3 OR S4 | 309,309 |
| S4 | (MH "Pulmonary Disease, Chronic Obstructive+") OR (TX "chronic respiratory disease*") OR (TX "obstructive lung disease*") OR TX COPD OR (TX emphysema*) OR TX "chronic Obstructive Pulmonary Disease" OR TX "Chronic Obstructive Airway Disease" OR TX "Chronic Obstructive Lung Disease" OR TX "pulmonary emphysema" OR TX "chronic bronchitis" OR TX COAD OR TX COBD OR TX AECB OR TX "Chronic Airflow Obstruction" | 27,917 |
| S3 | (MH "Osteoarthritis+") OR TX Osteoarthritis OR osteoarthr* OR (TX degenerative n1 TX arthritis) OR TX arthrosis | 34,765 |
| S2 | ( (MH "Diabetes Mellitus+") OR TX "Diabetes mellitus" OR TX "diabetes mellitus" OR TX diabetes OR TX "glucose intolerance" OR ( MM "insulin resistance" OR TX "insulin resistance") OR ( TX IDDM or TX NIDDM or TX MODY or TX T1DM or TX T2DM or TX T1D or TX T2D) OR TX "Diabetes Complications" NOT ( (MH "Diabetes Insipidus") OR TX "Diabetes Insipidus" ) | 217,465 |
| S1 | (MH "Asthma+") OR "asthma" | 37,457 |
