# Supplementary material for: Health Equity in the Effectiveness of Web-Based Health Interventions for the Self-Care of People With Chronic Health Conditions: Systematic Review
Source: J Med Internet Res. 2020 Jun 5;22(6):e17849. doi: 10.2196/17849 (PMC7305554; doi:10.2196/17849)
Supplement: Multimedia Appendix 2 [file jmir_v22i6e17849_app2.docx]

Table 2. Characteristics of included studies and populations*.*

| Author, Date | Study ID | Study details (study type, health condition, setting, and location) | Study sample size | Inclusion and exclusion criteria | Main outcome targeted by intervention | | Control/comparator |
| --- | --- | --- | --- | --- | --- | --- | --- |
| **Asthma** | | | | | | | |
| Kosse, 2019 [1] | A | Cluster RCT^a^;  asthma;  community pharmacies;  the Netherlands | 234 | Inclusion:  aged 12-18 years, active use of inhaled corticosteroid and/or beta-agonist, and  having a smartphone  Exclusion:  “insufficient comprehension of the Dutch language”, and those dependent on carers to take medication | Behavioral: improving medication adherence | Usual care—consisting of inhalation instruction and monitoring of prescriptions | |
| **COPD^b^** | | | | | | | |
| Moy, 2015 [2] | B | RCT;  COPD, emphysema, or chronic bronchitis;  Veterans from the United States and Puerto Rico identified from a national database of veterans;  United States | 239 | Inclusion:  aged ≥40 years; Could walk ≥1 block; Sedentary (<150 min of self-reported physical exercise per week); medical clearance from health provider; capacity to consent; checked email weekly; access to a computer with internet  Exclusion:  belong to veteran service networks signed up to a study using the intervention; enrolled in another pedometer-based walking program | Health: health-related QoL^c^  Behavioral: physical activity | Wait-list control subjects wore a pedometer every day but did not receive instructions about exercise or step-count goals and had access to a web page that only showed the study week. After the study, they were offered the intervention | |
| Voncken-Brewster, 2015 [3] | C | RCT;  COPD;  Recruited from a Dutch web-based panel assembled by Flycatcher internet research  the Netherlands | 1325 | Inclusion:  aged 40-70 years; proficient in Dutch; access to the internet; basic computer skills | Behavioral: smoking cessation and physical activity | Usual care | |
| **Diabetes** | | | | | | | |
| Bahar-Fuchs, 2019 [4] | D | RCT;  T2D^d^;  Recruited through media advertising, diabetes education groups, fliers distributed in local health centers, and through the Israel diabetes and cognitive decline observational study;  Israel | 84 | Inclusion:  fluency in Hebrew; Israeli resident in Tel-Aviv; Maccabi health services cover; Access to computer and internet at home; >6 years of education  Exclusion:  neurological/psychiatric disorder; medication for mood/cognition; visual/hearing impairment; challenges with daily activities; used cognitive training recently; in another clinical trial | Health: cognitive ability  Knowledge: self-management  Psychosocial: self-efficacy | No active control; described in Table 3 | |
| Davis, 2017 [5] | E | Single-arm pilot study;  T2D;  Recruited at a family medicine clinic and a pharmacy in eastern North Carolina;  United States | 51 | Inclusion:  aged ≥18 years; taking ≥1 medication for diabetes; English-speaking; nonadherent to medication; African American or white | Behavioral: medication adherence  Psychosocial: diabetes medicine self-efficacy | None | |
| Glasgow, 2012,[6] 2014[7] | F1  F2 | RCT;  T2D;  Primary care clinics within Kaiser Permanente Colorado selected based on variability in size, location, SES^e^ of neighborhood, and to maximize percentage of Latino patients;  United States | F1: 463  F2: 270 (secondary analysis of F1) | Inclusion:  aged 25-75 years; BMI >25 and risk factor for heart disease; access to telephone and internet; read and write English/Spanish; can perform mild to moderate physical activity | Health: blood pressure  Behavioral: (overall behavioral change): eating habits, fat intake, physical activity, and medical adherence  Psychosocial: self-efficacy | Enhanced usual care involved computer-based health risk feedback and recommendations for preventive care behaviors | |
| Heinrich, 2012 [8] | G | RCT;  T2D;  Conducted on the web;  the Netherlands | Not clearly given; n=135 used in analysis | Inclusion:  aged 40-70 years  Exclusion:  already used intervention | Knowledge: diabetes knowledge | Participants were allocated to the experimental group, the control group, or the post-test only control group. The latter was included to assess possible test effects of completing the pretest on post-test knowledge scores. Both control groups received access to the intervention after the post-test phase. | |
| Huang, 2014 [9] | H | RCT;  Chronic illness (inflammatory bowel disease, cystic fibrosis, and T1D^f^);  Tertiary care pediatric academic medical center;  United States | 81 | Inclusion:  aged 12-22 years; without cognitive impairment | Health: disease status  Behavioral: self- management  Psychosocial: self-efficacy | Controls received monthly messages via mail or email addressing general health issues. Usual health care communication was available to controls | |
| Istepanian, 2009 [10] | I | RCT;  T1D and T2D;  Thomas Addison Diabetes Centre at St George's Hospital in South London, United Kingdom, which serves a population with diverse ethnic mix of minority ethnic groups and a social deprivation score higher than national average;  United Kingdom | 137 | Inclusion:  aged >18 years; ambulant; diabetes and receiving treatment for hypertension/with an untreated blood pressure; capacity to consent  Exclusion:  physically unable to self-monitor blood glucose/pressure; pregnant; life-threatening illness | Health: blood pressure and blood glucose | Usual care | |
| Joubert, 2016 [11] | J | Prospective multicenter pilot study;  Pediatric departments in University hospitals;  France | 38 | Inclusion:  aged 11-18 years; diabetes ≥12 months; insulin therapy ≥6 months; prior usual care diabetes education  Exclusion:  celiac disease, corticosteroid; treatment, inflammatory disease; illiterate | Behavior: self-care  Knowledge: self-care knowledge | None | |
| Lorig, 2010 [12] | K | RCT;  T2D;  Recruitment via the internet, print, and broadcast media. Special effort was made to recruit American Indian/Alaskan native participants using websites and media associated with tribal and American Indian/Alaskan native organizations;  United States | 73 | Inclusion:  aged ≥18 years; not pregnant; not in care for cancer; access to the internet | Health: HbA_1c_^g^, fewer symptoms  Behavior: exercise  Psychosocial: health distress | Usual care. After 6 months, usual care participants in American Indian/Alaskan native subgroup were offered the program. All other usual care participants continued as control subjects through the 18 months of the study | |
| Offringa, 2018 [13] | L | Retrospective cohort;  T1 and T2D;  Participants were randomly selected from a database of diabetes users made up of people who had uploaded a blood glucose reading to their clinician’s office;  United States | 1799 | Inclusion:  uploaded their data two or more times between January 2011 and March 2017;  had at least 90 days of data before their initial upload  Exclusion:  erroneous meter readings | Health: average blood glucose  Behavioral: blood glucose testing rate | The control group was defined as any individual not using the mobile app, who uploaded their data at their health care practitioner’s office using the device as part of usual clinical care | |
| Pacaud, 2012 [14] | M | RCT;  T2D;  Recruited from the Building Healthy Lifestyles diabetes education program;  Canada | 79 | Inclusion:  newly diagnosed T2D; referred to the Building Healthy Lifestyles program; access to a computer with internet; computer literate; no complicating health conditions; not in another research study | Health: HbA_1c_  Knowledge: diabetes knowledge  Psychosocial: self-efficacy | Control group interacted with providers verbally and used an eHealth technology to support their structured diabetes education and their face-to-face learning task. They received paper-based educational materials and normal follow-up care. | |
| Raiff, 2016 [15] | N | RCT;  T1D;  Urban outpatient diabetes center;  United States | 52 | Inclusion:  aged 13-18 years; T1D ≥1 year; monitor blood glucose <4 a day; internet access at home; read and speak English  Exclusion:  did not attend diabetes clinic within 3 months of screening; disability that interfered with the study | Behavior: adherence with self-monitoring of blood glucose | Noncontingent submission participants were given the goal of performing and recording at least four blood glucose readings per day. Daily earnings for the noncontingent submission participant were determined by the contingent submission participant to whom they were matched | |
| Whittemore, 2012 [16] | P | RCT;  T1D;  pediatric diabetes clinics associated with Yale, The Children’s Hospital of Philadelphia, University of Arizona, and University of Miami representing a range of racial, ethnic, and socioeconomic backgrounds, reflective of the national prevalence of T1D in the youth;  United States | 320 | Inclusion:  aged 11-14 years; T1D for ≥6 months; age-appropriate school grade; not used coping skills training; no other significant health problem; access to high-speed internet; research staff assisted with internet access at school, library, or clinic where there was no access at home | Health: HbA_1c_  Psychosocial: quality of life | Managing diabetes was the attention control condition: 5 sessions were released weekly over 5 weeks. The sessions included case studies with culturally relevant content and problem-solving activities. The sessions were tailored and interactive | |
| Yu, 2014 [17] | Q | Single-arm pre-post cohort study;  T2D;  2 family practices and 2 endocrinology clinics in Toronto;  Canada | 81 | Inclusion:  aged ≥25 years;  at least one of: HbA_1c_ >7.0%, systolic blood pressure >130 mm Hg, low-density-lipoprotein cholesterol >2.0 mmol/L, or BMI > 25  Exclusion:  class 3 or 4 angina; did not speak English; not available for follow-up; no regular access to telephone and internet | Psychosocial: self-efficacy | None | |
| **Osteoarthritis** | | | | | | | |
| Lawford, 2018 [18] | R | RCT  Osteoarthritis  Recruited from the community  Australia | 148 | Inclusion:  aged ≥50 years; knee pain >3 months and most days of the previous month; knee pain during walking in the previous week, mild to moderate physical dysfunction; active email account; Computer with internet access | Health: walking pain, physical function | The control group received access to the same educational material as the intervention group, but did not have access to the pain coping skills training program | |
| Nevedal, 2013 [19] | S | One-group pretest posttest;  pain (including: joint, back, osteoarthritis, migraine, neuropathy);  Participants were employed by participating US companies or US health care plans that had purchased the web-based, digital pain management program (HealthMedia Inc Care for your Pain);  United States | 645 | Inclusion:  employed by participating US companies or a member of participating US health care plans | Health: pain experience (intensity, unpleasantness, and impact on daily activities)  Psychosocial: depression and self-efficacy | None | |

^a^RCT: randomized controlled trial.

^b^COPD: chronic obstructive pulmonary disease.

^c^QoL: quality of life.

^d^T2D: type 2 diabetes.

^e^SES: socioeconomic status.

^f^T1D: type 1 diabetes.

^g^HbA_1c_: hemoglobin A_1c_.

References included in the table:

1. Kosse RC, Bouvy ML, de Vries TW, Koster ES: **Effect of a mHealth intervention on adherence in adolescents with asthma: A randomized controlled trial**. *Respiratory Medicine* 2019, **149**:45-51.

2. Moy ML, Collins, Riley J., Martinez, Carlos H., Kadri, Reema, Roman, Pia, Holleman, Robert G., Kim, Hyungjin Myra, Nguyen, Huong Q., Cohen, Miriam D., Goodrich, David E., Giardino, Nicholas D., Richardson, Caroline R.: **An Internet-Mediated Pedometer-Based Program Improves Health-Related Quality-of-Life Domains and Daily Step Counts in COPD: A Randomized Controlled Trial**. *Chest* 2015, **148**(1):128-137.

3. Voncken-Brewster V, Tange, Huibert, de Vries, Hein, Nagykaldi, Zsolt, Winkens, Bjorn, van der Weijden, Trudy: **A randomized controlled trial evaluating the effectiveness of a web-based, computer-tailored self-management intervention for people with or at risk for COPD**. *International journal of chronic obstructive pulmonary disease* 2015, **10**:1061-1073.

4. Bahar-Fuchs A, Barendse ME, Bloom R, Ravona-Springer R, Heymann A, Dabush H, Bar L, Slater-Barkan S, Rassovsky Y, Schnaider Beeri M: **Computerized Cognitive Training for Older Adults at Higher Dementia Risk due to Diabetes: Findings From a Randomized Controlled Trial**. *The Journals of Gerontology: Series A* 2020, **75**(4):747-754.

5. Davis R, Campbell R, Hildon Z, Hobbs L, Michie S: **Theories of behaviour and behaviour change across the social and behavioural sciences: a scoping review**. *Health Psychology Review* 2015, **9**(3):323-344.

6. Glasgow RE, Kurz, Deanna, King, Diane, Dickman, Jennifer M., Faber, Andrew J., Halterman, Eve, Woolley, Tim, Toobert, Deborah J., Strycker, Lisa A., Estabrooks, Paul A., Osuna, Diego, Ritzwoller, Debra: **Twelve-month outcomes of an Internet-based diabetes self-management support program**. *Patient education and counseling* 2012, **87**(1):81-92.

7. Glasgow RE, Strycker, Lisa A., King, Diane K., Toobert, Deborah J.: **Understanding who benefits at each step in an internet-based diabetes self-management program: application of a recursive partitioning approach**. *Medical decision making : an international journal of the Society for Medical Decision Making* 2014, **34**(2):180-191.

8. Heinrich E, de Nooijer, Jascha, Schaper, Nicolaas C., Schoonus-Spit, Maartje H. G., Janssen, Monique A. J., de Vries, Nanne K.: **Evaluation of the web-based Diabetes Interactive Education Programme (DIEP) for patients with type 2 diabetes**. *Patient education and counseling* 2012, **86**(2):172-178.

9. Huang JS, Terrones, Laura, Tompane, Trevor, Dillon, Lindsay, Pian, Mark, Gottschalk, Michael, Norman, Gregory J., Bartholomew, L. Kay: **Preparing adolescents with chronic disease for transition to adult care: a technology program**. *Pediatrics* 2014, **133**(6):e1639-1646.

10. Istepanian RSH, Zitouni, Karima, Harry, Diane, Moutosammy, Niva, Sungoor, Ala, Tang, Bee, Earle, Kenneth A.: **Evaluation of a mobile phone telemonitoring system for glycaemic control in patients with diabetes**. *Journal of telemedicine and telecare* 2009, **15**(3):125-128.

11. Joubert M, Armand C, Morera J, Tokayeva L, Guillaume A, Reznik Y: **Impact of a serious videogame designed for flexible insulin therapy on the knowledge and behaviors of children with type 1 diabetes: the LUDIDIAB pilot study**. *Diabetes technology & therapeutics* 2016, **18**(2):52-58.

12. Lorig K, Ritter, Philip L., Laurent, Diana D., Plant, Kathryn, Green, Maurice, Jernigan, Valarie Blue Bird, Case, Siobhan: **Online diabetes self-management program: a randomized study**. *Diabetes care* 2010, **33**(6):1275-1281.

13. Offringa R, Sheng T, Parks L, Clements M, Kerr D, Greenfield MS: **Digital diabetes management application improves glycemic outcomes in people with type 1 and type 2 diabetes**. *Journal of diabetes science and technology* 2018, **12**(3):701-708.

14. Pacaud D, Kelley H, Downey AM, Chiasson M: **Successful Delivery of Diabetes Self-Care Education and Follow-Up through eHealth Media**. *Canadian Journal of Diabetes* 2012, **36**(5):257-262.

15. Raiff BR, Barrry VB, Ridenour TA, Jitnarin N: **Internet-based incentives increase blood glucose testing with a non-adherent, diverse sample of teens with type 1 diabetes mellitus: a randomized controlled Trial**. *Translational behavioral medicine* 2016, **6**(2):179-188.

16. Whittemore R, Jaser, Sarah S., Jeon, Sangchoon, Liberti, Lauren, Delamater, Alan, Murphy, Kathleen, Faulkner, Melissa S., Grey, Margaret: **An internet coping skills training program for youth with type 1 diabetes: six-month outcomes**. *Nursing research* 2012, **61**(6):395-404.

17. Yu CH, Parsons, Janet A., Mamdani, Muhammad, Lebovic, Gerald, Hall, Susan, Newton, David, Shah, Baiju R., Bhattacharyya, Onil, Laupacis, Andreas, Straus, Sharon E.: **A web-based intervention to support self-management of patients with type 2 diabetes mellitus: effect on self-efficacy, self-care and diabetes distress**. *BMC medical informatics and decision making* 2014, **14**:117.

18. Lawford BJ, Hinman RS, Kasza J, Nelligan R, Keefe F, Rini C, Bennell KL: **Moderators of effects of internet-delivered exercise and pain coping skills training for people with knee osteoarthritis: Exploratory analysis of the IMPACT randomized controlled trial**. *Journal of medical Internet research* 2018, **20**(5):e10021.

19. Nevedal DC, Wang, Chun, Oberleitner, Lindsay, Schwartz, Steven, Williams, Amy M.: **Effects of an individually tailored Web-based chronic pain management program on pain severity, psychological health, and functioning**. *Journal of medical Internet research* 2013, **15**(9):e201.
