# Supplementary material for: Health Equity in the Effectiveness of Web-Based Health Interventions for the Self-Care of People With Chronic Health Conditions: Systematic Review
Source: J Med Internet Res. 2020 Jun 5;22(6):e17849. doi: 10.2196/17849 (PMC7305554; doi:10.2196/17849)
Supplement: Multimedia Appendix 3 [file jmir_v22i6e17849_app3.docx]

**Table 3.** Participant characteristics.

| Study ID | Sample size | Age (years) | Sex (female), % | Ethnic group, % | Education | Employment, % | Income, % | Other PP^a^ category, % |
| --- | --- | --- | --- | --- | --- | --- | --- | --- |
| **Asthma** | | | | | | | | |
| A [1] | 253 | Mean 15.1 (SD 1.9) | 52.6 | Dutch: 88.5 | — | — | — | — |
| **COPD^b^** | | | | | | | | |
| B [2] | 239 | Mean 67 (SD 9.0) | 6.0 | — | — | — | — | Rural residence: 45.0 |
| C [3] | 1325 | Mean 57.6 (SD 7.2) | 52.0 | — | Primary/vocational school: 29.5%  Secondary vocational/high school degree: 32.7%  Higher professional/university degree: 37.8% | Employed: 51.3  Not employed: 48.7 | — | Marital status: single/divorced/widowed 26.6  In relationship/living together/married: 73.4 |
| D [4] | 84 | Mean 71.5 (SD 4.9) | 40.0 | — | Mean years of education 16 (SD 3.0) | — | — | — |
| **Diabetes** | | | | | | | | |
| E [5] | 51 | Mean 54.0 (SD 10.4) | 80.0 | Black/African American: 84.0  Other: 16 | — | — | — | — |
| F1[6] F2 [7] | F1: 463  F2: 219 | Mean 58.4 (SD 9.2) | 49.8 | AI/AN^c^: 6.7  Asian: 1.6  African American: 15.4  White: 72.0  Latino: 21.8 | High school: ≤19.1% | — | <$49,999: 47.3  $50,000-$89,999: 35.2  ≥$90,000: 17.5 | — |
| G [8] | 135 | **Experimental group (A)** | | | | | | |
|  |  | Mean 56.0 (SD 7.0) | 56.0 | — | Low education: 35.0%  Middle: 35.0%  High: 30.0% | — | — | — |
|  |  | **Control group (B)** | | | | | | |
|  |  | Mean 56.0 (SD 7.0) | 48.0 | — | Low education: 43.0%  Middle: 30.0%  High: 27.0% | — | — | — |
|  |  | **Control group (C)** | | | | | | |
|  |  | Mean 59.0 (SD 6.0) | 53.0 | — | Low education: 25.0%  Middle: 28.0%  High: 47.0% | — | — | — |
| H [9] | 81 | Median 17 (range 12-20) | 54.3 | White: 33.3  Black: 9.9  Hispanic: 37.0  AI/AN: 1.2  Other: 6.2 | — | — | — | — |
| I [10] | 137 | Mean 61.0 | — | White: 41.0  African Caribbean: 23.0  Indo-Asian: 35.0  Other: 1.0 | — | — | — | — |
| J [11] | 38 | Mean 13.7 (SD 2.1 ) | 58.0 | — | Primary school: 3.0%  College: 66.0%  High school: 32.0% | — | — | Parents married: 79.0  Parents separated: 21.0 |
| K [12] | 73 | Mean 50.5 (SD 8.7) | 80.0 | AI/AN: 100 | Mean years of education 15.6 (SD 2.56) | — | — | Married: 51.7 |
| L [13] | 1799 | Control: Mean 57.0 (SD 20.5)  Mobile: Mean 49.0 (SD 19.3) | Female: Control 0.2  Mobile 19.0  Unknown: Control 99.8  Mobile 32.0 | — | — | — | — | — |
| M [14] | 79 | Mean 54.2 (SD 9.1) | 52.9 | — | — | — | — | 75.6 lived in urban center |
| N [15] | 52 | Mean 15.20 (SD 1.43) | NC^d^: 44.4  CS^e^: 39.1 | White: NC 23.5, CS 21.7  Hispanic: NC 64.7, CS 60.9  African American: NC 11.8, CS 13.0  Multiracial: NC 0.0, CS 4.4 | — | — | Household income  <$30,000: NC 38.9, CS 26.1  $30,000–60,000: NC 16.6, CS 30.4  $60,000-100,000: NC 5.6, CS 13.1  >$100,000: NC 27.8, CS 26.0  Unknown: NC 11.1, CS 4.4 | — |
| P [16] | 320 | Mean 12.3 (SD 1.1) | 55.0 | White: 72.0 | — | — | ≥$80,000/year: 51.0% | — |
| Q [17] | 81 | 20-39 years: 9.0%  40-59 years: 46.0%  60-79 years: 44.0%  >80 years: 1.0% | 46.0 | White: 62.0  Asian: 30.0  African American: 7.0  Hispanic: 1.0 | High school: <1.0%  High school: 14.0%  College: 26.0%  University: 59.0% | Employed: 56.0  Retired: 30.0  Unemployed: 9.0  Disability: 2.0  Student: 4.0 | < Can $15,000 (US $10,899  ): 21.0  Can $15,000- 29,999 (US $10,899-21,798): 10.0  Can $30,000-59,999 (US $ 21,799-43,597): 27.0  ≥Can $60,000 (US $43,598): 28.0 | — |
| **Osteoarthritis** | | | | | | | | |
| R [18] | 148 | Intervention: Mean 60.8 (SD 6.5)  Control: Mean 61.5 (SD 7.6) | 56.1 | — | No tertiary training: Intervention 22%, Control 32%  Some tertiary training: Intervention 78%, Control 68% | Currently employed either full-time or part-time: 57.4 | — | — |
| S [19] | 645 | 22-29 years: 2.2%  30-39 years: 8.4%  40-49 years: 17.2%  50-59 years: 33.5%  60-69 years: 23.1%  70-91 years: 15.7% | 69.3 | White: 78.8  African American: 8.4  Hispanic: 5.9  Other: 6.9 | — | Professional: 18.7  Clerical/administrative: 21.7  Not working outside home: 31.5  Sales/technical support: 15.7  Executive/senior manager: 5.9  Production/laborer: 6.6 | — | — |

^a^PP: PROGRESS plus.

^b^COPD: chronic obstructive pulmonary disease.

^c^AI/AN: American Indian/Alaskan Native.

^d^NC: noncontingent group.

^e^CS: contingent group.

Empty cells represent missing data.

References included in the table:

1. Kosse RC, Bouvy ML, de Vries TW, Koster ES: **Effect of a mHealth intervention on adherence in adolescents with asthma: A randomized controlled trial**. *Respiratory Medicine* 2019, **149**:45-51.

2. Moy ML, Collins, Riley J., Martinez, Carlos H., Kadri, Reema, Roman, Pia, Holleman, Robert G., Kim, Hyungjin Myra, Nguyen, Huong Q., Cohen, Miriam D., Goodrich, David E., Giardino, Nicholas D., Richardson, Caroline R.: **An Internet-Mediated Pedometer-Based Program Improves Health-Related Quality-of-Life Domains and Daily Step Counts in COPD: A Randomized Controlled Trial**. *Chest* 2015, **148**(1):128-137.

3. Voncken-Brewster V, Tange, Huibert, de Vries, Hein, Nagykaldi, Zsolt, Winkens, Bjorn, van der Weijden, Trudy: **A randomized controlled trial evaluating the effectiveness of a web-based, computer-tailored self-management intervention for people with or at risk for COPD**. *International journal of chronic obstructive pulmonary disease* 2015, **10**:1061-1073.

4. Bahar-Fuchs A, Barendse ME, Bloom R, Ravona-Springer R, Heymann A, Dabush H, Bar L, Slater-Barkan S, Rassovsky Y, Schnaider Beeri M: **Computerized Cognitive Training for Older Adults at Higher Dementia Risk due to Diabetes: Findings From a Randomized Controlled Trial**. *The Journals of Gerontology: Series A* 2020, **75**(4):747-754.

5. Davis R, Campbell R, Hildon Z, Hobbs L, Michie S: **Theories of behaviour and behaviour change across the social and behavioural sciences: a scoping review**. *Health Psychology Review* 2015, **9**(3):323-344.

6. Glasgow RE, Kurz, Deanna, King, Diane, Dickman, Jennifer M., Faber, Andrew J., Halterman, Eve, Woolley, Tim, Toobert, Deborah J., Strycker, Lisa A., Estabrooks, Paul A., Osuna, Diego, Ritzwoller, Debra: **Twelve-month outcomes of an Internet-based diabetes self-management support program**. *Patient education and counseling* 2012, **87**(1):81-92.

7. Glasgow RE, Strycker, Lisa A., King, Diane K., Toobert, Deborah J.: **Understanding who benefits at each step in an internet-based diabetes self-management program: application of a recursive partitioning approach**. *Medical decision making : an international journal of the Society for Medical Decision Making* 2014, **34**(2):180-191.

8. Heinrich E, de Nooijer, Jascha, Schaper, Nicolaas C., Schoonus-Spit, Maartje H. G., Janssen, Monique A. J., de Vries, Nanne K.: **Evaluation of the web-based Diabetes Interactive Education Programme (DIEP) for patients with type 2 diabetes**. *Patient education and counseling* 2012, **86**(2):172-178.

9. Huang JS, Terrones, Laura, Tompane, Trevor, Dillon, Lindsay, Pian, Mark, Gottschalk, Michael, Norman, Gregory J., Bartholomew, L. Kay: **Preparing adolescents with chronic disease for transition to adult care: a technology program**. *Pediatrics* 2014, **133**(6):e1639-1646.

10. Istepanian RSH, Zitouni, Karima, Harry, Diane, Moutosammy, Niva, Sungoor, Ala, Tang, Bee, Earle, Kenneth A.: **Evaluation of a mobile phone telemonitoring system for glycaemic control in patients with diabetes**. *Journal of telemedicine and telecare* 2009, **15**(3):125-128.

11. Joubert M, Armand C, Morera J, Tokayeva L, Guillaume A, Reznik Y: **Impact of a serious videogame designed for flexible insulin therapy on the knowledge and behaviors of children with type 1 diabetes: the LUDIDIAB pilot study**. *Diabetes technology & therapeutics* 2016, **18**(2):52-58.

12. Lorig K, Ritter, Philip L., Laurent, Diana D., Plant, Kathryn, Green, Maurice, Jernigan, Valarie Blue Bird, Case, Siobhan: **Online diabetes self-management program: a randomized study**. *Diabetes care* 2010, **33**(6):1275-1281.

13. Offringa R, Sheng T, Parks L, Clements M, Kerr D, Greenfield MS: **Digital diabetes management application improves glycemic outcomes in people with type 1 and type 2 diabetes**. *Journal of diabetes science and technology* 2018, **12**(3):701-708.

14. Pacaud D, Kelley H, Downey AM, Chiasson M: **Successful Delivery of Diabetes Self-Care Education and Follow-Up through eHealth Media**. *Canadian Journal of Diabetes* 2012, **36**(5):257-262.

15. Raiff BR, Barrry VB, Ridenour TA, Jitnarin N: **Internet-based incentives increase blood glucose testing with a non-adherent, diverse sample of teens with type 1 diabetes mellitus: a randomized controlled Trial**. *Translational behavioral medicine* 2016, **6**(2):179-188.

16. Whittemore R, Jaser, Sarah S., Jeon, Sangchoon, Liberti, Lauren, Delamater, Alan, Murphy, Kathleen, Faulkner, Melissa S., Grey, Margaret: **An internet coping skills training program for youth with type 1 diabetes: six-month outcomes**. *Nursing research* 2012, **61**(6):395-404.

17. Yu CH, Parsons, Janet A., Mamdani, Muhammad, Lebovic, Gerald, Hall, Susan, Newton, David, Shah, Baiju R., Bhattacharyya, Onil, Laupacis, Andreas, Straus, Sharon E.: **A web-based intervention to support self-management of patients with type 2 diabetes mellitus: effect on self-efficacy, self-care and diabetes distress**. *BMC medical informatics and decision making* 2014, **14**:117.

18. Lawford BJ, Hinman RS, Kasza J, Nelligan R, Keefe F, Rini C, Bennell KL: **Moderators of effects of internet-delivered exercise and pain coping skills training for people with knee osteoarthritis: Exploratory analysis of the IMPACT randomized controlled trial**. *Journal of medical Internet research* 2018, **20**(5):e10021.

19. Nevedal DC, Wang, Chun, Oberleitner, Lindsay, Schwartz, Steven, Williams, Amy M.: **Effects of an individually tailored Web-based chronic pain management program on pain severity, psychological health, and functioning**. *Journal of medical Internet research* 2013, **15**(9):e201.
