# Supplementary material for: Health Equity in the Effectiveness of Web-Based Health Interventions for the Self-Care of People With Chronic Health Conditions: Systematic Review
Source: J Med Internet Res. 2020 Jun 5;22(6):e17849. doi: 10.2196/17849 (PMC7305554; doi:10.2196/17849)
Supplement: Multimedia Appendix 4 [file jmir_v22i6e17849_app4.docx]

**Table 4.** Intervention description.

| Study ID | Type of intervention | | Intervention content | | External technology or support | | Duration of intervention and follow-up | Attrition (number without outcome, number randomizedn/N %) | |
| --- | --- | --- | --- | --- | --- | --- | --- | --- | --- |
| **Asthma** | | | | | | | | | |
| A [1] | App | The app was developed using the common sense model of self-regulation. It was connected to the patient’s community pharmacist and had elements to target nonadherence to medication: (1) CARAT^a^ to monitor disease control over time; (2) short educational and motivational videos; (3) medication reminder alarm; (4) peer and pharmacist chat; (5) questions to monitor nonadherence to medication | | Pharmacists could monitor CARAT scores, send additional videos, contact the patient, and change the app settings | | Intervention: 6 months  Follow-up: 6 months | | | 19/253 (7.5%) |
| **COPD^b^** | | | | | | | | | |
| B [2] | Website | THS^c^ was an internet-mediated, pedometer-based walking program to promote physical activity based on self-regulation theory. Tailored algorithms based on the data provided dynamic individualized incremental walking goals and feedback about success at meeting goals. THS also include education on disease self-management, motivational support, and an online community for social support | | None | | Intervention: 12 months  Follow-up: 4-month | | | 13/239 (5.5%) |
| C [3] | App | *MasterYourBreath* was a web-based application providing computer-generated tailored feedback based on the i-Change model. This model includes the Attitude-Social influence-Self-efficacy model, which incorporates ideas of the Theory of Planned Behaviour, the Social Cognitive Theory, the Trans-theoretical Model, the Health Belief Model, and Implementation and Goal setting theories. The app had 2 behavior change, smoking cessation, and physical activity modules that provided feedback on: (1) behavior based on Dutch guidelines; (2) perceived positive and negative consequences of behavior; (3) the social influences on the behavior. There was goal setting and action plans; self-efficacy and maintenance feedback to maintain the healthy behavior. Feedback was personalized using participants’ names and tailored to participants’ characteristics and key behavior determinants of psychosocial constructs | | None | | Intervention: 6 months  Follow-up: 6-month follow-up | | | 254/1325 (19.16%) |
| **Diabetes** | | | | | | | | | |
| D [4] | Website | There were 2 interventions: (1) tailored and adaptive CCT^d^, (2) generic CCT. Within the cognitive training conditions, participants were further randomized into a global self-efficacy, cognitive training self-efficacy, or a no self-efficacy condition. All participants trained at home on the CogniFit platform and received psychoeducation and a range of BCTs^e^ used to optimize adherence to the CCT intervention. Behavioral components of the interventions were developed using Michie et al’s taxonomy of BCTs and theoretical domains framework.[5] | | The self-efficacy condition included fortnightly support phone calls and 2 video clips sent via email to support self-efficacy. Participants in the *no self-efficacy condition* received a standard phone monitoring call every 2 weeks, without self-efficacy–specific BCTs | | Intervention:  8-week Follow-up: 6-month | | | 8/84 (9%) |
| E[6] | Website | The intervention included nine 2-min video intervention modules that discussed strategies for dealing with diabetes-related issues. The participants read a list of 9 problems using diabetes medications, based on empirical studies, medication-related skills and adherence, and according to the information-motivation-behavioral skills model. The computer listed the problem areas the patient had identified, and patients were then asked to prioritize the order they wanted to watch the videos, they watched the first video module on their list during the visit. Participants were given access to a website so that they could watch the rest of the video modules | | The research assistant called the patient at 1, 4, and 8 weeks to ask what changes they made after watching the videos | | Intervention:  Not clearly specified  Follow-up: 3 months | | | 16/51 (31%) |
| F1  [7] and F2 [8] | Website | The 2 interventions were developed using social cognitive theory and a social ecological model. (1) CASM^f^: Participants selected daily goals, recorded progress, and received feedback on reaching goals. There was a moderated forum and community resources. The website graphically displayed the participant’s HbA_1c_^g^, blood pressure, and cholesterol results. After 6 weeks, participants created new personalized goals, identified barriers to achieving the goal(s), and chose from a list of problem-solving strategies.  (2) CASM+SS^g^: CASM program plus 2 phone calls from an interventionist, and an invitation to attend 3 group visits | | None | | Intervention: Not clearly specified  Follow-up: 4 months and 12 months | | | 105/463  (22.7%) |
| G [9] | Website | The Diabetes interactive education program gave an overview of T2D^h^ management in 7 chapters. Information was provided on basic and more advanced levels. Each chapter closed with questions to the patient and had a workbook with goal-setting forms, checklists on self-management behaviors, and space to note down questions for their clinician. The team did not reference any theory for intervention development | | None | | Intervention: 2 weeks  Follow-up: 2 weeks | | | 31/166 (18.7%) |
| H [10] | Website | MD2ME was developed using Bandura’s social cognitive theory. MD2ME monitored disease symptoms, responded to monitoring with appropriate treatments, and worked with clinicians to manage care. Subjects logged in to a website weekly to receive materials outlining common disease management, communication skills, and lifestyle tips. Disease-specific case studies were provided. An automated SMS algorithm provided disease management decision support, and a health care team communications portal. Subjects could use SMS to report health concerns which were relayed to the health care team depending on urgency | | None | | Intervention: 8 months  Follow-up: 2 months and 8 months | | | 6/81 (7%) |
| I [11] | App | Mobile health was structured into 3 main elements: (1) blood glucose and blood pressure monitoring with Bluetooth connectivity. Recordings were performed weekly. The mobile phone provided a prompt when a measurement was due; (2) The web interface and patients journals that could be accessed by the nurse or the physician; (3) Web-based patient journal that allowed the patient to interact with the nurse. The team did not reference any theory for intervention development | | Blood glucose monitor with wireless transfer | | Intervention: Not clearly specified  Follow-up: Recorded weekly up until 9 months | | | Only numbers at randomization given |
| J  [12] | Website | A web-based video game, *serious game*. Social cognitive and self-determination theories provided key mediating variables on which to focus a serious video game promoting self-management: knowledge, skills, self-efficacy, and motivation. The game involved problem-solving issues around T1D management. The player has to manage the T1D of Alex (main character). The player adapts a glycemic simulator and insulin dose injection for each meal according to Alex’s parameters. This game was not customizable to the type of treatment (insulin pump/multiple daily injections) or the age of the player | | None | | Intervention:6 months  Follow-up: 2 weeks and 6 months | | | 9/47 (19%) |
| K  [13] | Website | There were 2 versions of the intervention: (1) IDSMP^i^, (2) IDSMP with email reinforcement. IDSMP was based on English- and Spanish-language peer-led small-group diabetes self-management programs. It consisted of 6 weekly sessions, and included: learning center (questions and actions); discussion center: with bulletin boards populated by responses made in the learning center and new threads started by participants; *My Tools* with exercise and medication logs, audio relaxation exercises, meal planning, and glucose-monitoring tools and links to other diabetes-related websites; *Post Office*: participants and facilitators could write private, messages to each other. The team did not reference any theory for intervention development | | A book, Living a Healthy Life with Chronic Conditions. Sections of the book were referenced in the learning center | | Intervention: 6 weeks  Follow-up: The subgroup analysis was reported at 6-month follow-up only | | | 0/45 (0%) |
| L [14] | App | The mobile app contained a digital logbook, user-initiated blood glucose data capture, and graphical displays of the glucose data. Mobile users also had the ability to view stored blood glucose data in graphical format, to complement glucose data with personal information about food intake, medications, and exercise, and the ability to set reminders for themselves to support their care plan. The team did not reference any theory for intervention development | | None | | Intervention:3 months  Follow-up: 6 months | | | 0/1799 (0.00%) |
| M [15] | Website | There were 2 interventions: (1) web static: email with providers and a second type of eHealth^j^ technology (eg, electronic blood glucose journal) to support their electronic learning. They received follow-up care with providers by email.  (2) Web interactive: used email and private and public chats with providers and other patients and use of a third type of eHealth technology (electronic blood glucose journal and additional functional, eg, bulletin board) to support their learning and to access education and tools. They received normal care via virtual appointments with providers using email and private chats. The team did not reference any theory for intervention development | | None | | Intervention: Not clear, mentioned 3 months to 12 months in original BHL^k^ program  Follow-up: 3, 6, 9, and 12-months | | | 11/79 (14%) |
| N [16] | Website | The intervention was based on contingency management and motivational interviewing strategies. Contingent submission treatment condition—participants were told that to earn vouchers they needed to submit SMBG^l^ videos that showed them meeting their daily goals. The first 3 days served as a shaping period where the goals were gradually increased, starting with a goal of at least 1 sample on the first day, 2 on the second, and 3 on the third. From day 4, participants were required to submit ≥4 SMBG during a 24-hour period to earn vouchers, separated by at least 2 hours per test. Each test resulted in an immediate $1.00 voucher and they could earn vouchers for up to 8 tests per day, with a $3.00 bonus for the fourth video of the day. Participants could exchange their vouchers at any time for gift cards at preferred vendors | | Participants received one brief motivational interviewing session at the end of recruitment, which lasted around 20 min and was delivered by a clinician or research assistant trained by a certified instructor | | Intervention: 20 days  Follow-up: 40 days | | | 11/52 (21%) |
| P[17] | Website | TEENCOPE used a graphic novel format and a cast of ethnically diverse characters with T1D who present challenging social situations, approaches to solving problems, and consequences of decisions. TEENCOPE was based on a successful in-person coping skills training program for youth with T1D and included: social skills training, cognitive behavior modification, assertive communication, stress reduction, and conflict resolution; 5 sessions were released weekly that were interactive and encouraged self-assessment and the use of coping skills. At the end of each session, participants were asked to practice the new coping skills and to share experiences on a discussion board moderated by a health professional | | None | | Intervention: 5 weeks  Follow-up: (1) 3 months and 6 months follow-up  (1a) 3 months, 6 months, and 12 months follow-up | | | Psychosocial 103/320 (32.2%)  Health outcome 48/320 (15.0%) |
| Q [17] | Website | A diabetes web-based companion was developed using self-efficacy theory that incorporated evidence-based content and behavior change strategies and followed the principles of user-centered design. It consisted of 4 main components: (1) general information (static); (2) tailored information (interactive); (3) self-monitoring logs (interactive), and; (4) blog (interactive). Initially one blog was posted a week, after 4 weeks of limited user activity, this was increased to 2 per week and email prompts were added with each new posting. Participants received weekly email prompts to visit the site, complete their self-management trackers and notices of new content | | None | | Intervention: Not clear  Follow-up: Questionnaires were obtained every 3 weeks for 9 months | | | 8/81 (10%) |
| **Osteoarthritis** | | | | | | | | | |
| R [18] | Website | The intervention was developed using social cognitive theory and adult learning theory. Participants received 3 internet-delivered treatments: (1) educational material about exercise and physical activity, pain management, emotions, healthy eating, complementary therapies, and medications; (2) Web-based interactive automated pain coping skills training program and; (3) 7 physical therapist consultations via Skype. A female virtual *coach* spoke directly to the user, providing verbal instruction, feedback, and encouragement. COACH- track promoted self-monitoring by allowing participants to review and change goals, record practices and *coping confidence* (self-efficacy), view graphic summaries of their progress, and manage automated reminders. COACH-chat allowed them to read about others’ experiences and to submit descriptions of their own experiences. | | Physical therapist consultations via Skype. Over 12 weeks, with each consultation lasting 30-45 min. Physical therapists performed a brief assessment and prescribed a lower limb strengthening home exercise program to be completed by the participant 3 times per week. Participants were also given a pedometer if desired. | | Intervention: 3 months  Follow-up: 3 and 9 months | | | 16/148 (10.8%) |
| S [19] | Website | HealthMedia Inc Care for your Pain integrated evidence-based theories of cognitive behavioral treatment, chronic disease self-management, motivational enhancement, and theories of health behavior change, including social cognitive theory, theory of reasoned action, theory of planned behavior, and self-determination theory. The program was commercially available (not directly to consumers) and used patient self-report data and algorithms developed by expert clinicians to provide tailored information and interventions based on participant’s pattern of responses to an interactive consultation | | None | | Intervention: Not clear  Follow-up: 6 months | | | Not clearly stated |

^a^CARAT: control of allergic rhinitis and asthma test.

^b^COPD: chronic obstructive pulmonary disease.

^c^THS: Taking Healthy Steps.

^d^CCT: computerized cognitive training.

^e^BCT: behavior change techniques.

^f^CASM: computer-assisted self-management.

^g^HbA_1c_: hemoglobin A_1c_.

^h^SS: Social Support

^i^T2D: type 2 diabetes.

^j^IDSMP: internet-based diabetes self-management program.

^k^eHealth: electronic health.

^l^SMBG: self-management blood glucose.

References included in the table:

1. Kosse RC, Bouvy ML, de Vries TW, Koster ES: **Effect of a mHealth intervention on adherence in adolescents with asthma: A randomized controlled trial**. *Respiratory Medicine* 2019, **149**:45-51.

2. Moy ML, Collins, Riley J., Martinez, Carlos H., Kadri, Reema, Roman, Pia, Holleman, Robert G., Kim, Hyungjin Myra, Nguyen, Huong Q., Cohen, Miriam D., Goodrich, David E., Giardino, Nicholas D., Richardson, Caroline R.: **An Internet-Mediated Pedometer-Based Program Improves Health-Related Quality-of-Life Domains and Daily Step Counts in COPD: A Randomized Controlled Trial**. *Chest* 2015, **148**(1):128-137.

3. Voncken-Brewster V, Tange, Huibert, de Vries, Hein, Nagykaldi, Zsolt, Winkens, Bjorn, van der Weijden, Trudy: **A randomized controlled trial evaluating the effectiveness of a web-based, computer-tailored self-management intervention for people with or at risk for COPD**. *International journal of chronic obstructive pulmonary disease* 2015, **10**:1061-1073.

4. Bahar-Fuchs A, Barendse ME, Bloom R, Ravona-Springer R, Heymann A, Dabush H, Bar L, Slater-Barkan S, Rassovsky Y, Schnaider Beeri M: **Computerized Cognitive Training for Older Adults at Higher Dementia Risk due to Diabetes: Findings From a Randomized Controlled Trial**. *The Journals of Gerontology: Series A* 2020, **75**(4):747-754.

5. Michie S, Richardson M, Johnston M, Abraham C, Francis J, Hardeman W, Eccles MP, Cane J, Wood CE: **The behavior change technique taxonomy (v1) of 93 hierarchically clustered techniques: building an international consensus for the reporting of behavior change interventions**. 2013(1532-4796 (Electronic)).

6. Davis R, Campbell R, Hildon Z, Hobbs L, Michie S: **Theories of behaviour and behaviour change across the social and behavioural sciences: a scoping review**. *Health Psychology Review* 2015, **9**(3):323-344.

7. Glasgow RE, Kurz, Deanna, King, Diane, Dickman, Jennifer M., Faber, Andrew J., Halterman, Eve, Woolley, Tim, Toobert, Deborah J., Strycker, Lisa A., Estabrooks, Paul A., Osuna, Diego, Ritzwoller, Debra: **Twelve-month outcomes of an Internet-based diabetes self-management support program**. *Patient education and counseling* 2012, **87**(1):81-92.

8. Glasgow RE, Strycker, Lisa A., King, Diane K., Toobert, Deborah J.: **Understanding who benefits at each step in an internet-based diabetes self-management program: application of a recursive partitioning approach**. *Medical decision making : an international journal of the Society for Medical Decision Making* 2014, **34**(2):180-191.

9. Heinrich E, de Nooijer, Jascha, Schaper, Nicolaas C., Schoonus-Spit, Maartje H. G., Janssen, Monique A. J., de Vries, Nanne K.: **Evaluation of the web-based Diabetes Interactive Education Programme (DIEP) for patients with type 2 diabetes**. *Patient education and counseling* 2012, **86**(2):172-178.

10. Huang JS, Terrones, Laura, Tompane, Trevor, Dillon, Lindsay, Pian, Mark, Gottschalk, Michael, Norman, Gregory J., Bartholomew, L. Kay: **Preparing adolescents with chronic disease for transition to adult care: a technology program**. *Pediatrics* 2014, **133**(6):e1639-1646.

11. Istepanian RSH, Zitouni, Karima, Harry, Diane, Moutosammy, Niva, Sungoor, Ala, Tang, Bee, Earle, Kenneth A.: **Evaluation of a mobile phone telemonitoring system for glycaemic control in patients with diabetes**. *Journal of telemedicine and telecare* 2009, **15**(3):125-128.

12. Joubert M, Armand C, Morera J, Tokayeva L, Guillaume A, Reznik Y: **Impact of a serious videogame designed for flexible insulin therapy on the knowledge and behaviors of children with type 1 diabetes: the LUDIDIAB pilot study**. *Diabetes technology & therapeutics* 2016, **18**(2):52-58.

13. Lorig K, Ritter, Philip L., Laurent, Diana D., Plant, Kathryn, Green, Maurice, Jernigan, Valarie Blue Bird, Case, Siobhan: **Online diabetes self-management program: a randomized study**. *Diabetes care* 2010, **33**(6):1275-1281.

14. Offringa R, Sheng T, Parks L, Clements M, Kerr D, Greenfield MS: **Digital diabetes management application improves glycemic outcomes in people with type 1 and type 2 diabetes**. *Journal of diabetes science and technology* 2018, **12**(3):701-708.

15. Pacaud D, Kelley H, Downey AM, Chiasson M: **Successful Delivery of Diabetes Self-Care Education and Follow-Up through eHealth Media**. *Canadian Journal of Diabetes* 2012, **36**(5):257-262.

16. Raiff BR, Barrry VB, Ridenour TA, Jitnarin N: **Internet-based incentives increase blood glucose testing with a non-adherent, diverse sample of teens with type 1 diabetes mellitus: a randomized controlled Trial**. *Translational behavioral medicine* 2016, **6**(2):179-188.

17. Yu CH, Parsons, Janet A., Mamdani, Muhammad, Lebovic, Gerald, Hall, Susan, Newton, David, Shah, Baiju R., Bhattacharyya, Onil, Laupacis, Andreas, Straus, Sharon E.: **A web-based intervention to support self-management of patients with type 2 diabetes mellitus: effect on self-efficacy, self-care and diabetes distress**. *BMC medical informatics and decision making* 2014, **14**:117.

18. Lawford BJ, Hinman RS, Kasza J, Nelligan R, Keefe F, Rini C, Bennell KL: **Moderators of effects of internet-delivered exercise and pain coping skills training for people with knee osteoarthritis: Exploratory analysis of the IMPACT randomized controlled trial**. *Journal of medical Internet research* 2018, **20**(5):e10021.

19. Nevedal DC, Wang, Chun, Oberleitner, Lindsay, Schwartz, Steven, Williams, Amy M.: **Effects of an individually tailored Web-based chronic pain management program on pain severity, psychological health, and functioning**. *Journal of medical Internet research* 2013, **15**(9):e201.
