# Supplementary material for: Health Equity in the Effectiveness of Web-Based Health Interventions for the Self-Care of People With Chronic Health Conditions: Systematic Review
Source: J Med Internet Res. 2020 Jun 5;22(6):e17849. doi: 10.2196/17849 (PMC7305554; doi:10.2196/17849)
Supplement: Multimedia Appendix 5 [file jmir_v22i6e17849_app5.docx]

Table 5: Overview of available data for social characteristics modifying intervention effects

| Condition | Number of studies where there is evidence of modification of intervention effect by PP variable/ Total number of studies that explored modification of intervention effect | What PP interactions did they look at? | Outcome | Evidence of an interaction | Interactions found |
| --- | --- | --- | --- | --- | --- |
|  |  |  |  |  |  |
| Asthma | 1/1 | Age,  Gender | Behavioural | Yes | + High score on Medication Adherence Report Scale (MARS) x age (mean) in study A: The patients who scored in the higher range for MARS( >19) were on average 0.7 years older (P=.02) in adolescents aged 12–18 years. No effect was found for gender (P=.88) [1] |
| COPD | 1/2 | Age  Gender  Education  Employment  Urban/rural | Health | No | 0 QoL x Age in veterans age ≥ 40 years old in study B. No other social characteristics were investigated.[2] |
|  |  |  | Behavioural | Yes | 0 Physical activity x Age, gender, education, employment adults aged between 40–70 years of age in study C.[3]  + Daily step count x Age(increasing) in Study B: Each 1-year increase in age was associated with a 33-point decrease in change in daily step counts (P = .03) in US army veterans age ≥ 40 years old. No other participant characteristics were investigated [2] |
|  |  |  | Knowledge | ? | No interactions explored |
|  |  |  | Psychosocial | ? | No interactions explored |
| Diabetes | 11/13 | Age  Gender  Ethnicity  Education  Health Literacy  Numeracy  Income  Family structure | Health | Yes | 0 HbA1c x age, gender, ethnicity, income in youth aged 11-14 years with T1D in study P.[4]  0 Blood Glucose level x gender in adults (no age limit given) with T1 and T2D in study L.[5]  + HbA1c x Gender (male) in study M: trend for a group by time by gender interaction for change in A1C from baseline to the final visit was found (F[2, 62]=2.76, p=0.071). Males in the Web Interactive group and the Web Static group tended to drop in A1C by the final visit, but males in the control group showed a slight increase in A1C from the first to final visit (F[2, 29]=3.05, p=0.063).) No associations were found for women in a study of people with T2D(no age limit given). No other interactions with social characteristics were explored.[6]  - Cognition x gender (male) study D: being a female was associated with having a “clinically meaningful” improvement in cognition (>=0.5 SD) (β = −1.90, stderr = 0.87, P = .03) in older adults (no age specified). 0 Cognition x age and education.[7]  + Blood pressure x Ethnicity (minority group) study F1: Latinos had a greater reduction in blood pressure than non-Latinos in the intervention arm, while Latinos had a lower reduction in the control arm (no estimates available p=.006). But 0 Blood pressure x Age, gender, education, health literacy and numeracy in adults with T2D aged 25-75 years at 12 months.[8]  + Blood pressure x Ethnicity (minority group) in study I: mean decrement in blood pressure in adults >18years with T1 or T2D was greater within the patients of African-Caribbean heritage, compared with the Caucasians and Indo-Asians in the intervention arm. The mean SBP increased for the Caucasians and to a much lesser extent, the African- Caribbean’s in the control arm. The Indo-Asian’s mean SBP decreased in both intervention group and to a greater extent in the control group (no estimates provided). No other interactions with social characteristics investigated.[9] |
|  |  |  | Behavioural | Yes | 0 Patient initiated communication x Age, health literacy in adolescents with T1D IBD and CF aged 12-20 years transitioning into adult care study H.[10]  0 Overall behavioural change x Age, gender, Latino ethnicity, education, health literacy or numeracy in adults with T2D aged 25-75 years at 12 months in study F1.[8]  0 Maintenance of behaviour x Age, gender, Latino ethnicity, health literacy, education, numeracy in adults with T2D aged 25-75 years at 12 months in F2.[11]  0 blood glucose testing rate x gender in adults (no age limit given) with T1 and T2D in study L.[5]  0 Self-care x Gender in people with T2D(no age limit given) study M. [6]  +Self-care x age (increasing) in study Q: improvement was positively correlated with age (0.04/year, 95% CI: 0.02, 0.06), p <0.001). 0 self-care x gender, ethnicity, education, employment, income or health literacy in people aged ≥ 25 years with T2D.[12]  +Blood glucose testing rate x age in study N: older adolescents responded (16–18) better to having treatment (1.04 more daily glucose tests, P<.01) than younger adolescents(13–15) in a group of adolescents with T1D aged 13–18 [13]  + Activity Limitation x Ethnicity(minority group) in study K: strong association between decrease in activity limitation for AI/AN program participants compared with the control group in adults with T2D aged ≥18 years (effect size 0.337, P (ITT) 0.028, P=.012). No other interactions with PP groups investigated [14]  +Self-management x education in study D: more years of education (β = 0.28, stand err = 0.12, p = .03) were associated with being a respondent on the self-management outcome in older adults (no age specified).No association with age and gender.[7] |
|  |  |  | Knowledge | Yes | 0 Diabetes knowledge x Age, gender or education in adults with T2D aged 40–70 years. [15] / gender in people with T2D(no age limit given) study M [6]  + Chronic disease management knowledge x Health Literacy(high) in study H: in adolescent participants with T1D IBD and CF aged 12-20 years in the intervention arm with ‘adequate literacy levels’ demonstrated greater improvements in disease management knowledge over the study period compared with those with ‘inadequate literacy levels’ (no estimates provided). But 0 Chronic disease management knowledge x Age.[10]  + Diabetes self-care knowledge x Family structure(divorced parents) in study N: a higher proportion of the children from the high effect subgroup had divorced parents, compared with the low effect subgroup (35% vs. 6%, respectively; P < 0.05) in children aged age 11–18 years with T1D.. 0 x age, gender and education.[16] |
|  |  |  | Psychosocial | Yes | 0 Self-efficacy x Health literacy, age in adolescents with T1D IBD and CF aged 12-20 years transitioning into adult care in study H.[10]  0 Self-efficacy x age, gender, Latino ethnicity, education, health literacy or numeracy in adults with T2D aged 25-75 years at 12 months in study F1.[8]  0 Self-efficacy x gender in people with T2D (no age limit given) study M.[6]  + Diabetes distress x age (increasing), gender (male) in study Q : improvements in diabetes distress following the use of the intervention was greater for older (estimate -0.34, SE 0.15, 95%CI -0.64 - -0.05, p=0.01) males (gender(male) estimate -9.12, SE 3.62, 95% CI -16.22 - -2.02, P=.006) participants aged ≥ 25 years with T2D. 0 Diabetes distress x ethnicity, education, employment, income or health literacy.[12]  + Health distress x ethnicity(minority group) study K: strong association between decrease in health distress for AI/AN program participants compared with the control group in adults with T2D aged ≥18 years (P=.003).[57]  + Diabetes QoL x Ethnicity (minority ethnic group) in study P: Participants of black/other ethnicity benefitted more from the TEENCOPE intervention than white, non-Hispanic/non-Latino participants (3.01 ± 1.25 vs. 0.28 ± 0.85; P = .07). But 0 Diabetes QoL x age, gender or income in youth aged 11-14 years with T1D.[4]  + Diabetes medication self-efficacy x health literacy in study E: patients with a Rapid Estimate of Adult Literacy in Medicine (REALM) for health literacy (REALM) score of eighth grade or lower experienced a significantly greater improvement in their diabetes medication self-efficacy scores than patients who read at ninth grade or higher (t = 2.54, P = .02) in adults aged 18 or older.[17]. 0 diabetes medication self-efficacy x age, gender and ethnicity. |
| Osteoarthritis | 1/2 | Age,  gender, ethnicity,  education,  employment,  people living in their household | Health | Yes | 0 treatment responders (pain composite of pain intensity and unpleasantness reduced to a clinically meaningful level (average pain reduction ≥ 30%)x Age, gender, ethnicity, or number of people living in the household in working age adults opting in to a web-based pain management programme (no age range given) in study S.[18]  Two primary health outcomes for Lawford, 2018:  + Walking pain x employment status in study R: There was some evidence for moderation of the treatment effect by employment status for change in walking pain at 3 months in adults ≥50 years (interaction P=.02). Among those who were currently employed, participants assigned to the intervention group had greater reductions in pain than those in the control group, with an estimated difference between groups of mean 2.38 (95% CI 1.52-3.23) NRS units. Among unemployed participants, the estimated difference in reduction in pain at 3 months between the intervention and control groups was mean 0.86 (–0.13 to 1.85) NRS units. There was no evidence of a difference at 9 months. 0 Walking pain x age, gender or education.[19]  0 Physical function x Age, gender, education and employment at 3 or 9 months in adults ≥50 years.[19] |

References included in the table:

1. Kosse RC, Bouvy ML, de Vries TW, Koster ES: **Effect of a mHealth intervention on adherence in adolescents with asthma: A randomized controlled trial**. *Respiratory medicine* 2019, **149**:45-51.

2. Moy ML, Collins RJ, Martinez CH, Kadri R, Roman P, Holleman RG, Kim HM, Nguyen HQ, Cohen MD, Goodrich DE *et al*: **An Internet-Mediated Pedometer-Based Program Improves Health-Related Quality-of-Life Domains and Daily Step Counts in COPD: A Randomized Controlled Trial**. *Chest* 2015, **148**(1):128-137.

3. Voncken-Brewster V, Tange H, de Vries H, Nagykaldi Z, Winkens B, van der Weijden T: **A randomized controlled trial evaluating the effectiveness of a web-based, computer-tailored self-management intervention for people with or at risk for COPD**. *International journal of chronic obstructive pulmonary disease* 2015, **10**:1061-1073.

4. Whittemore R, Jaser SS, Jeon S, Liberti L, Delamater A, Murphy K, Faulkner MS, Grey M: **An internet coping skills training program for youth with type 1 diabetes: six-month outcomes**. *Nursing research* 2012, **61**(6):395-404.

5. Offringa R, Sheng T, Parks L, Clements M, Kerr D, Greenfield MS: **Digital Diabetes Management Application Improves Glycemic Outcomes in People With Type 1 and Type 2 Diabetes**. *Journal of diabetes science and technology* 2018, **12**(3):701-708.

6. Pacaud D, Kelley H, Downey AM, Chiasson M: **Successful Delivery of Diabetes Self-Care Education and Follow-Up through eHealth Media**. *Canadian Journal of Diabetes* 2012, **36**(5):257-262.

7. Bahar-Fuchs A, Barendse MEA, Bloom R, Ravona-Springer R, Heymann A, Dabush H, Bar L, Slater-Barkan S, Rassovsky Y, Schnaider Beeri M: **Computerized Cognitive Training for Older Adults at Higher Dementia Risk due to Diabetes: Findings From a Randomized Controlled Trial**. *The Journals of Gerontology: Series A* 2019.

8. Glasgow RE, Kurz D, King D, Dickman JM, Faber AJ, Halterman E, Woolley T, Toobert DJ, Strycker LA, Estabrooks PA *et al*: **Twelve-month outcomes of an Internet-based diabetes self-management support program**. *Patient Education & Counseling* 2012, **87**(1):81-92 12p.

9. Istepanian RSH, Sungoor A, Earle KA: **Technical and compliance considerations for mobile health self-monitoring of glucose and blood pressure for patients with diabetes**. *Conference proceedings : Annual International Conference of the IEEE Engineering in Medicine and Biology Society IEEE Engineering in Medicine and Biology Society Annual Conference* 2009, **2009**:5130-5133.

10. Huang JS, Terrones L, Tompane T, Dillon L, Pian M, Gottschalk M, Norman GJ, Bartholomew LK: **Preparing adolescents with chronic disease for transition to adult care: a technology program**. *Pediatrics* 2014, **133**(6):e1639-1646.

11. Glasgow RE, Strycker LA, King DK, Toobert DJ: **Understanding who benefits at each step in an internet-based diabetes self-management program: application of a recursive partitioning approach**. *Medical decision making : an international journal of the Society for Medical Decision Making* 2014, **34**(2):180-191.

12. Yu CH, Parsons JA, Mamdani M, Lebovic G, Hall S, Newton D, Shah BR, Bhattacharyya O, Laupacis A, Straus SE: **A web-based intervention to support self-management of patients with type 2 diabetes mellitus: effect on self-efficacy, self-care and diabetes distress**. *BMC medical informatics and decision making* 2014, **14**:117.

13. Raiff BR, Barrry VB, Ridenour TA, Jitnarin N: **Internet-based incentives increase blood glucose testing with a non-adherent, diverse sample of teens with type 1 diabetes mellitus: a randomized controlled Trial**. *Translational behavioral medicine* 2016, **6**(2):179-188.

14. Lorig K, Ritter PL, Laurent DD, Plant K, Green M, Jernigan VB, Case S: **Online diabetes self-management program: a randomized study**. *Diabetes care* 2010, **33**(6):1275-1281 1277p.

15. Heinrich E, de Nooijer J, Schaper NC, Schoonus-Spit MH, Janssen MA, de Vries NK: **Evaluation of the web-based Diabetes Interactive Education Programme (DIEP) for patients with type 2 diabetes**. *Patient Education & Counseling* 2012, **86**(2):172-178 177p.

16. Joubert M, Armand C, Morera J, Tokayeva L, Guillaume A, Reznik Y: **Impact of a Serious Videogame Designed for Flexible Insulin Therapy on the Knowledge and Behaviors of Children with Type 1 Diabetes: The LUDIDIAB Pilot Study**. *Diabetes technology & therapeutics* 2016, **18**(2):52-58.

17. Davis SA, Carpenter D, Cummings DM, Lee C, Blalock SJ, Scott JE, Rodebaugh L, Ferreri SP, Sleath B: **Patient adoption of an internet based diabetes medication tool to improve adherence: A pilot study**. *Patient education and counseling* 2017, **100**(1):174-178.

18. Nevedal DC, Wang C, Oberleitner L, Schwartz S, Williams AM: **Effects of an individually tailored Web-based chronic pain management program on pain severity, psychological health, and functioning**. *J Med Internet Res* 2013, **15**(9):e201.

19. Lawford BJ, Hinman RS, Kasza J, Nelligan R, Keefe F, Rini C, Bennell KL: **Moderators of Effects of Internet-Delivered Exercise and Pain Coping Skills Training for People With Knee Osteoarthritis: Exploratory Analysis of the IMPACT Randomized Controlled Trial**. *Journal of medical Internet research* 2018, **20**(5):e10021.
