# Supplementary material for: Health Equity in the Effectiveness of Web-Based Health Interventions for the Self-Care of People With Chronic Health Conditions: Systematic Review
Source: J Med Internet Res. 2020 Jun 5;22(6):e17849. doi: 10.2196/17849 (PMC7305554; doi:10.2196/17849)
Supplement: Multimedia Appendix 6 [file jmir_v22i6e17849_app6.docx]

Table 6: Modification of effectiveness data available for PROGRESS- Plus categories in the included studies

| **Study** | **Study id** | **Health condition** | **Study size** | **Study type** | **RoB** | **Outcome** | **Age (increasing)** | **Gender (male)** | **Ethnicity (minority)** | **Education (high)** | **Employed** | **Income (higher)** | **Numeracy (high)** | **Health literacy (high)** | **Number of people in household** | **Family structure (divorced parents)** |
| --- | --- | --- | --- | --- | --- | --- | --- | --- | --- | --- | --- | --- | --- | --- | --- | --- |
|  |  |  |  |  |  |  |  |  |  |  |  |  |  |  |  |  |
| Kosse, 2019 | A | Asthma | 234 | Cluster RCT | High | Behavior | + | 0 |  |  |  |  |  |  |  |  |
| Moy, 2015 | B | COPD | 239 | RCT | Low | Health | 0 |  |  |  |  |  |  |  |  |  |
|  |  |  |  |  |  | Behavior | _ |  |  |  |  |  |  |  |  |  |
| Voncken-Brewster, 2015 | C | COPD | 1325 | RCT | High | Behavior | 0 | 0 |  | 0 | 0 |  |  |  |  |  |
| Bahar-Fuchs, 2019 | D | Diabetes | 84 | RCT | Low | Health | 0 | - |  | 0 |  |  |  |  |  |  |
|  |  |  |  |  |  | Behavior | 0 | 0 |  | + |  |  |  |  |  |  |
| Davis, 2017 | E | Diabetes | 51 | Single arm pilot study | Critical | Psychosocial | 0 | 0 | 0 |  |  |  |  | - |  |  |
| Glasgow, 2012 | F1 | Diabetes | 463 | RCT | High | Health | 0 | 0 | + | 0 |  |  | 0 | 0 |  |  |
|  |  |  |  |  |  | Behavior | 0 | 0 | 0 | 0 |  |  | 0 | 0 |  |  |
|  |  |  |  |  |  | Psychosocial | 0 | 0 | 0 | 0 |  |  | 0 | 0 |  |  |
| Glasgow, 2014 | F2 | Diabetes | 463 | RCT | High | Behavior | 0 | 0 | 0 | 0 |  |  | 0 | 0 |  |  |
| Heinrich, 2012 | G | Diabetes | 135 | RCT | Unclear | Knowledge | 0 | 0 |  | 0 |  |  |  |  |  |  |
| Huang, 2014 | H | Diabetes | 81 | RCT | Unclear | Behavior | 0 |  |  |  |  |  |  | 0 |  |  |
|  |  |  |  |  |  | Knowledge | 0 |  |  |  |  |  |  | + |  |  |
|  |  |  |  |  |  | Psychosocial | 0 |  |  |  |  |  |  | 0 |  |  |
| Istepanian, 2009 | I | Diabetes | 137 | RCT | High | Health |  |  | + |  |  |  |  |  |  |  |
| Joubert, 2016 | J | Diabetes | 38 | Prospective multi-center pilot study | Critical | Knowledge | 0 | 0 |  | 0 |  |  |  |  |  | + |
| Lorig, 2010 | K | Diabetes | 73 | RCT | Unclear | Behavior |  |  | + |  |  |  |  |  |  |  |
|  |  |  |  |  |  | Psychosocial |  |  | + |  |  |  |  |  |  |  |
| Offringa, 2018 | L | Diabetes | 1799 | Retrospective cohort | Critical | Health |  | 0 |  |  |  |  |  |  |  |  |
|  |  |  |  |  |  | Behavior |  | 0 |  |  |  |  |  |  |  |  |
| Pacaud, 2012 | M | Diabetes | 79 | RCT | High | Health |  | + |  |  |  |  |  |  |  |  |
|  |  |  |  |  |  | Behavior |  | 0 |  |  |  |  |  |  |  |  |
|  |  |  |  |  |  | Knowledge |  | 0 |  |  |  |  |  |  |  |  |
|  |  |  |  |  |  | Psychosocial |  | 0 |  |  |  |  |  |  |  |  |
| Raiff, 2016 | N | Diabetes | 52 | RCT | High | Behavior | + |  |  |  |  |  |  |  |  |  |
| Whittemore,2012 | P | Diabetes | 320 | RCT | Low | Health | 0 | 0 | 0 |  |  | 0 |  |  |  |  |
|  |  |  |  |  |  | Psychosocial | 0 | 0 | + |  |  | 0 |  |  |  |  |
| Yu, 2014 | Q | Diabetes | 81 | Non-RCT | Critical | Behavior | + | 0 | 0 | 0 | 0 | 0 |  | 0 |  |  |
|  |  |  |  |  |  | Psychosocial | + | + | 0 | 0 | 0 | 0 |  | 0 |  |  |
| Lawford, 2018 | R | Osteoarthritis | 148 | RCT | Low | Health (R1-Walking pain) | 0 | 0 |  | 0 | + |  |  |  |  |  |
|  |  |  |  |  |  | Health (R2- Physical function) | 0 | 0 |  | 0 | 0 |  |  |  |  |  |
| Nevedal, 2013 | S | Osteoarthritis | 645 | Non-RCT | Critical | Health | 0 | 0 | 0 |  |  |  |  |  | 0 | 0 |

+ Positive association

0 No association

- Negative association
